# Diverse reactivity of the gem-difluorovinyl iodonium salt for direct incorporation of the difluoroethylene group into N- and O-nucleophiles

**DOI:** 10.1038/s42004-022-00772-7

**Published:** 2022-12-03

**Authors:** Chenxin Ge, Bin Wang, Yunchen Jiang, Chao Chen

**Affiliations:** 1 Key Laboratory of Bioorganic Phosphorus & Chemical Biology (Ministry of Education, MOE), Tsinghua University, 100084 Beijing, China; 2 State Key Laboratory of Elemento-Organic Chemistry, Nankai University, Tianjin, 300071 China

**Keywords:** Synthetic chemistry methodology, Drug discovery and development, Synthetic chemistry methodology

## Abstract

The synthesis of gem-difluoroethylene compounds remains a difficult task in organic synthesis. Here, the direct difluoroethylation reactions of N- and O-nucleophiles including amides and acids were realized with a hypervalent iodine reagent: gem-difluorovinyl iodonium salt (DFVI). The reactions were accomplished via a neighbouring group rearrangement. The gem-difluorovinyl iodonium salt was found to display diverse reactivity due to its unique electronic effect and was applied to the incorporation of difluoroethylene group, including difluorovinylation of carboxylic acids, difluorovinylation and 1,3-cyclic fluorovinylation of amides and 1,1-cyclic difluoroethylation of amines.

## Abstract (web summary)

The gem-difluoroethylene moiety is considered as a valuable building block in organic synthesis, but methods to introduce it into small molecules are generally limited to specific substrates. Here, the authors demonstrate that a gem-difluorovinyl iodonium salt enables the direct difluoroethylation reactions of a diverse range of N- and O-nucleophiles.

## Introduction

**Fig. 1:**
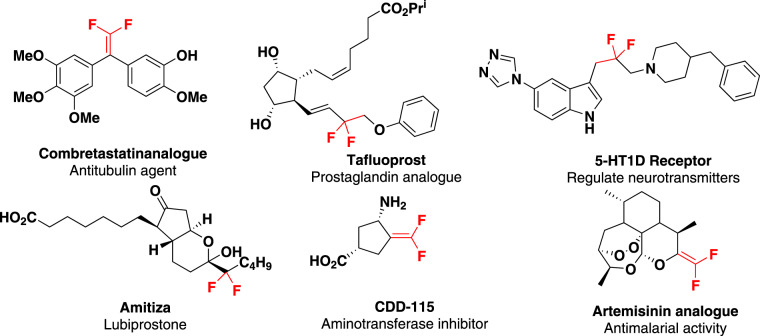
Presence of gem-difluoroethylene moiety. gem-Difluoroethylene moieties present in natural products and potential therapeutic compounds.

The incorporation of fluorine has been known to change the organic molecules’ physical, chemical and biological properties significantly. Therefore, organofluorine compounds have attracted widespread attention in various fields such as agricultural chemistry^1^, medicinal chemistry^2–4^, and material sciences^5–7^. As an important component of fluorine-containing groups, gem-difluoroethylene moiety (CH_2_CF_2_) is considered as stable bioisostere for metabolically susceptible keto group in drug research^8–10^. The replacement of a carbonyl group with a gem-difluoroethylene moiety improves the in vivo absorption rate and reduce the in vivo metabolism (Fig. 1)^8,11^. As a valuable building block in organic synthesis, it has been applied to a multitude of organic transformations, including defluorosilylation^12^, defluoroborylation^13,14^, defluoro cross-coupling with alkyl halides^15^, [3 + 2] annulation^16–18^, hydrothiolation to access α,α-difluoroalkylthioethers and so on.

More recently, many efficient methods for the introduction of gem-difluoro olefin group into small molecules have been developed, but these methods are often limited to some specific substrates. For example, classic synthetic methods for the incorporation of gem-difluorovinyl moiety include gem-difluoroolefination of carbonyl compounds^19–22^ such as the Wittig reaction, the Julia–Kocienski reaction, and the Horner–Wadsworth–Emmons reaction. α-trifluoromethyl substituted alkene or carbene surrogates are also found to be efficient precursors via the C-X (X = C, B, O) bond formation subsequently C-F elimination^23–29^. Apart from this, the strategy that cross-coupling reaction of difluorocarbene with other carbene precursors has been successfully demonstrated^30–32^. Undoubtedly, the most convenient and direct way to introduce gem-difluoro olefin is to employ gem-difluorovinyl reagent. But until now, gem-difluorovinyl reagents are too rare to meet the needs of organic synthesis from the synthetic chemist. To the best of our knowledge, the electrophilic gem-difluorovinyl reagents reported hitherto are mainly limited to gem-difluorovinyl iodide and gem-difluorovinyl sulfonate. The gem-difluorovinyl iodide is gaseous leading to operation with difficulty and usually needs to be prepared as an organometallic reagent in advance to improve reactivity^33–35^. The gem-difluorovinyl sulfonate suffers from harsh reaction conditions or limited substrate scope^36–39^.

**Fig. 2:**
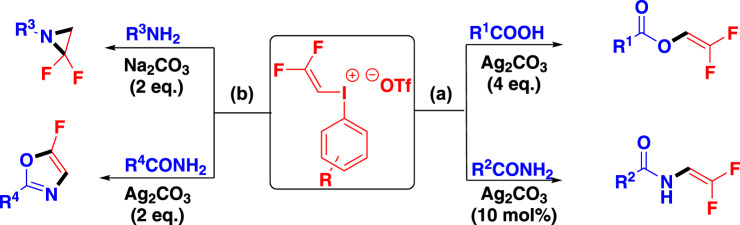
The structure and reaction modes of novel gem-difluorovinyl hypervalent iodine reagent (DFVI). **a** gem-Difluorovinyl iodonium salt acted as electrophiles. **b** gem-Difluorovinyl iodonium salt acted as bielectrophiles.

As a continuation of our interest in both hypervalent iodine reagents and fluorine-containing chemistry^40–48^, herein we like to report a new type of gem-difluorovinyl hypervalent iodine reagent (DFVI). Different from usual alkenyl groups, gem-difluorovinyl iodonium salt has strong electrophilicity due to the strong induction of fluorine atom and trivalent iodonium ion, thus it can react with O- and N-nucleophiles such as carboxylic acid and amide to afford gem-difluorovinyl ester and amide which are otherwise hardly accessible (Fig. 2). Furthermore, the increase of the amounts of silver carbonate in the reaction, the resulting difluorovinyl amide could perform intramolecular ring closure to generate fluorine-substituted oxazole with amide acting as 1,3-N,O-binucleophiles and gem-difluorovinyl iodonium salt acting as 1,2-bielectrophiles. By analogy, replacing the amides by amines (1,1-N,N-binucleophiles), gem-difluoro aziridine rings could be constructed. The above results have enriched the reaction mode of alkenyl iodonium salts^49,50^ and provided a novel method for introducing gem-difluorovinyl group.

## Results and discussion

### Synthesis of gem-difluorovinyl iodonium salt

**Fig. 3:**
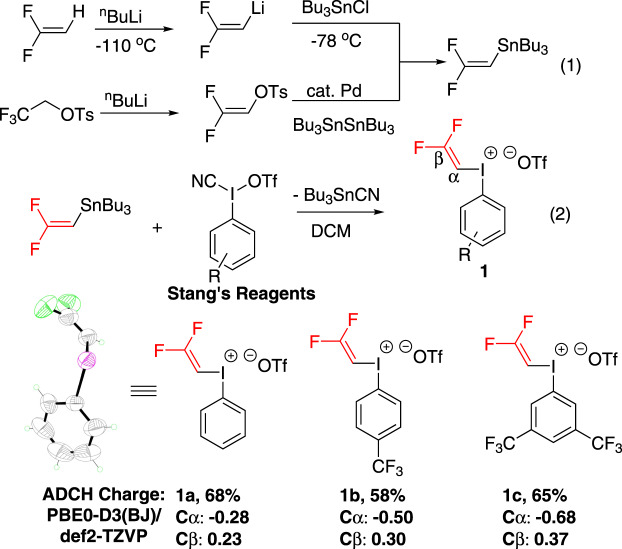
The synthesis and ADCH charge of gem-difluorovinyl iodonium reagents (DFVI) 1. X-ray structure of **1a** (CCDC No.: 2141360). The counter anion is omitted for clarity.

In an attempt to prepare the electrophilic gem-difluorovinyl iodonium salt **1**, we initially tried the reaction of cyano(aryl)-λ^3^-iodanyl trifluoromethanesulfonate with tributyl(2,2-difluorovinyl)stannane^51^ in DCM from −40 °C to room temperature. we were pleased to find that versatile gem-difluorovinyl iodonium salt **1a**–**1c** were successfully synthesized with corresponding Stang’s reagents (Fig. 3). It was worth mentioning that in the preparation of gem-difluorovinyl iodonium salt **1a**, the proposal that cyano(phenyl)-λ^3^-iodanyl trifluoromethanesulfonate intermediate was purified by employing a catheter tied with filter paper to drain the solvent below 0 °C was adopted due to the instability of the corresponding Stang’s reagent^52^. The desired gem-difluorovinyl iodonium salt **1a**–**1c** were obtained in 58–68% yield, as determined by ^1^H, ^13^C, ^19^F NMR spectroscopy. The gem-difluorovinyl iodonium salt structure was unambiguously confirmed by single crystal X-ray diffraction analysis of **1a** (Supplementary Table S1). The preparation method could be scaled up to 20 mmol scale without decreasing the yield. In addition, iodonium salt **1a** could be stored at 0 °C in the refrigerator for at least 1 year without degradation (characterized by ^1^H NMR spectroscopy). The calculation revealed the neat charge of −0.28 on Cα and 0.23 on Cβ in **1a**. The substitution of CF_3_ group on the aryl ring of DFVI would significantly change the neat charge of double bond and reached to −0.68 on Cα and 0.37 on Cβ in **1c** (Fig. 3). This unique charge distribution could affect the reactivity of DFVI (vide infra).

### gem-Difluorovinylation of carboxylic acid

**Fig. 4:**
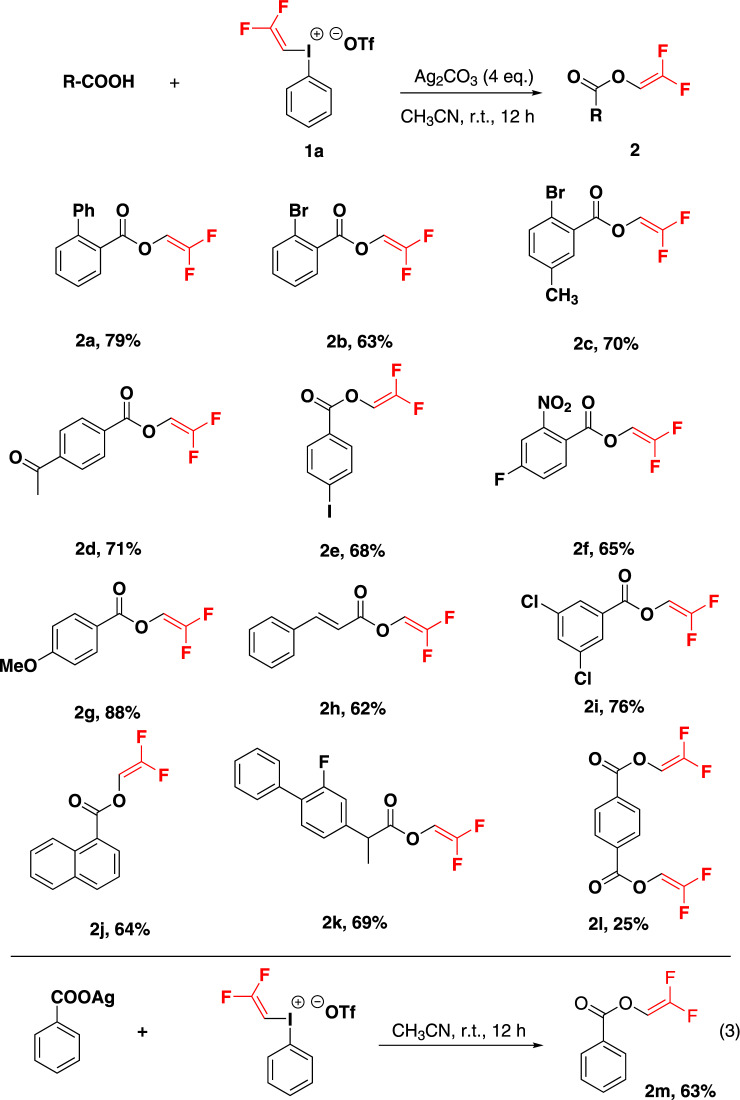
Electrophilic gem-difluorovinylation of carboxylic acid. Reaction conditions: carboxylic acid (0.2 mmol), **1a** (0.8 mmol), Ag_2_CO_3_ (0.8 mmol) in CH_3_CN (2.0 ml) at room temperature for 12 h. Yields shown are of isolated products.

With this new DFVI reagent in hand, we then explored its application in the incoporation of CHCF_2_ moiety with a variety of nucleophiles. Oxygen nucleophiles such as carboxylic acid compounds, were found to react smoothly with **1a** to give the corresponding gem-difluorovinyl esters when Ag_2_CO_3_ was used as the base (Fig. 4). Generally, aryl-, alkenyl- and benzyl-substituted carboxylic acid reacted gently to give gem-difluorovinyl carboxylate **2** in moderate to good yields. The electron-withdrawing groups substituted on the phenyl ring, such as phenyl, bromine, carbonyl, iodine groups, even strong electron-withdrawing nitro group had no significant effects on the yields. Besides, electron-donating groups substituted on the phenyl ring gave the desired product in good yield (**2g**). 3,5-dichlorobenzoic acid, 1-naphthoic acid were also excellent substrates, leading to gem-difluorovinyl carboxylate (**2i**, **2j**). To illustrate potential utility, the methodology was applied to the gem-difluorovinylation of selected drug. For instance, flurbiprofen, an anti-inflammatory drug used to treat rheumatoid arthritis, osteoarthritis, could also be converted to the corresponding gem-difluorovinyl carboxylate product **2k** in 69% yield. Although para-bienol esters **2l** were obtained in relatively low yield, they were valuable starting materials in polymer chemistry. Considering the alkyl and aryl carboxylic acid *pKa* was ~4.2, and the carbonate *pKa1* was 6.2, we envisioned whether the reaction underwent silver carboxylate intermediate. To verify this, we combined silver benzoate with gem-difluorovinyl iodonium salt **1a** at 1:1 molar ratio, 2,2-difluorovinyl benzoate was achieved in 63% yield [Eq. (3)].

### gem-Difluorovinylation of amide

Enamides are versatile building blocks for the synthesis of nitrogen-containing molecules. As stable and electron-rich olefins, they participate in diverse transformations such as asymmetric hydrogenation^53^, cycloaddition^54,55^, and C–H activation^56,57^. Inspired by the successful results with carboxylic acid, we next studied reactions with amides, as so far there was a still no-good method to synthesize gem-difluoro enamides. Under the reaction conditions similar to carboxylic acid, however there was no gem-difluorovinyl amide generated. Fluorine-substituted oxazole was the major product which would be described in the following text. Gratifyingly, when we used the catalytic amount of Ag_2_CO_3_, main product of gem-difluorovinyl amide was observed accompanied by starting materials. Nevertheless, the conversion rate of **1a** was very low, ~50%. When we attempted to use bases (e.g. Na_2_CO_3_ and DTBPy) to capture the trifluoromethanesulfonic acid produced in the system to improve the conversion rate, yet the yield of desired product did not increase, and side product trifluoroethyl amide was produced much. In view of the weak alkalinity of amide, we tried to make amide itself as base and increased its amount to twice that of the gem-difluoro iodonium salt. To our delight, we found that gem-difluorovinyl iodonium salt was converted completely thus the yield of title compound was increased to 68%. Further screening of the amount of amide led to the yield of the desired product reaching to 82%. Under the optimized reaction conditions, the substrate scope was studied.

**Fig. 5:**
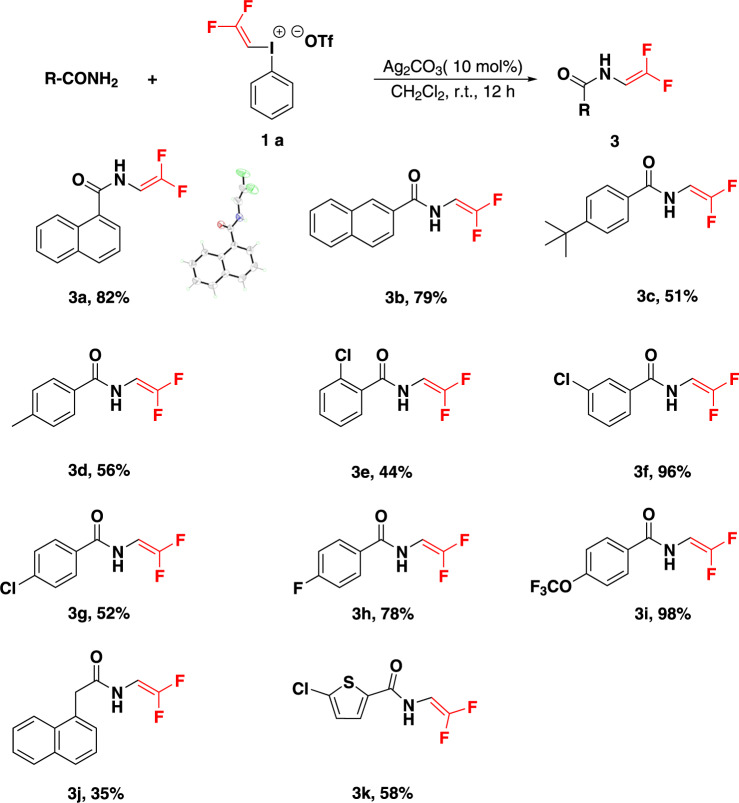
Electrophilic gem-difluorovinylation of amide. Reaction conditions: amide (0.8 mmol), **1a** (0.2 mmol), Ag_2_CO_3_ (0.02 mmol) in CH_2_Cl_2_ (2.0 ml) at room temperature for 12 h. Yields shown are of isolated products. CCDC No.: 2141364.

As shown in Fig. 5, both electron-rich and electron-deficient amides reacted to give the corresponding products in high yields. Various functional groups, including methyl, fluorine, tert-butyl, chlorine, trifluoromethoxy groups, were all tolerated under the standard conditions. Notably, polycyclic aryl and benzyl amide were also compatible with reaction conditions (**3a**–**3b**, **3j**). Reactions of heteroaryl amide occurred smoothly to give the gem-difluorovinyl products in moderate yields (**3k**). NMR spectra obtained from the mixture of amide with gem-difluorovinyl iodonium salt in CD_2_Cl_2_ showed a significant upfield shift (0.19 ppm) of the F resonance in iodonium salt **1a** and simultaneously a downfield shift (0.34 ppm) of the active N proton signal in amide (see SI). Both observations provided evidence for weak interaction between iodonium salt and nucleophile reagent amide. The gem-difluorovinyl structure was unambiguously confirmed by single crystal X-ray diffraction analysis of **3a** (Supplementary Table S2).

### Fluorovinylation of amide

**Fig. 6:**
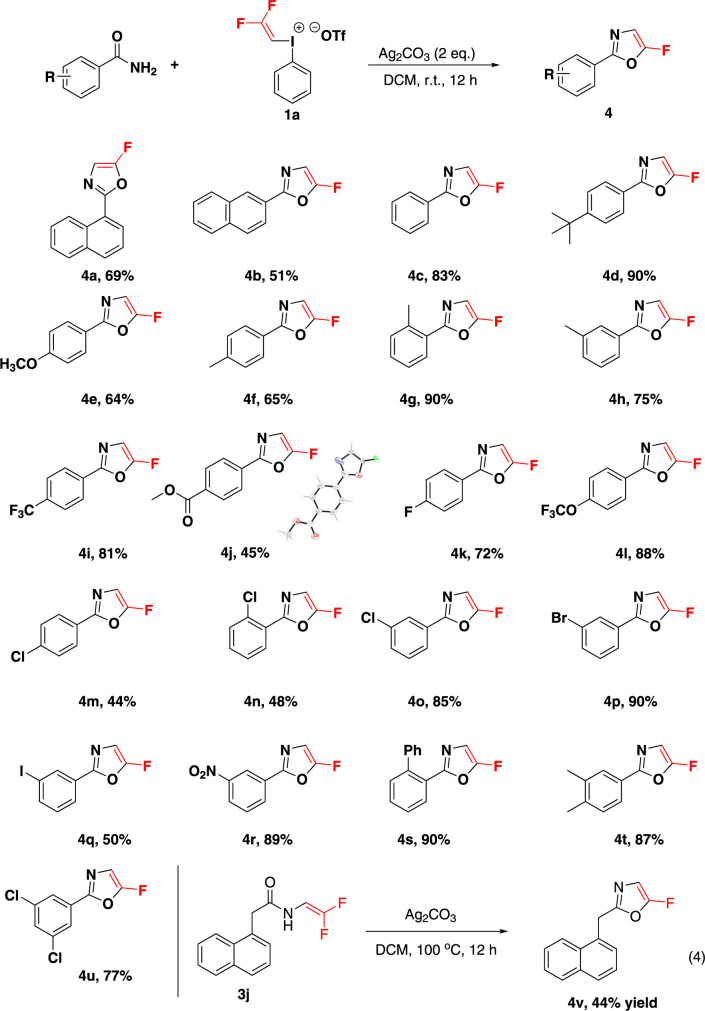
Electrophilic fluorovinylation of amide. Reaction conditions: amide (0.2 mmol), **1a** (0.24 mmol), Ag_2_CO_3_ (0.4 mmol) in CH_2_Cl_2_ (2.0 ml) at room temperature for 12 h. Yields shown are of isolated products. CCDC No.: 2141361.

As mentioned above, due to the leaving ability of fluorine atom in gem-difluorovinyl group, 1,1 gem-difluorovinyl amides could be further transformed into oxazoles through intramolecular ring closure. After a quick screening of the reaction conditions, it turned out that reactions conducted with Ag_2_CO_3_ (200% mol) in DCM resulted in full conversion of amides into fluoro oxazoles (Fig. 6). After establishing the optimum reaction conditions, we set out to evaluate the substrate scope. To our delight, both electron-donating and -withdrawing groups were well tolerated and the corresponding products **4a**–**4u** were furnished in good to high yields. Excellent compatibility of various functionalities including alkyl, methoxy, ester, trifluoromethyl, trifluoromethoxy, fluorine, chlorine, bromine, iodine, nitro was observed (**4d**–**4s**). In addition, this protocol was also applicable for bisubstituted and polycyclic aryl amides as starting materials affording the products (**4a**–**4b**, **4t**–**4u**) with good efficiency. The fluorine-substituted oxazole structure was unambiguously confirmed by single crystal X-ray diffraction analysis of **4j** (Supplementary Table S3). To gain insight into the reaction mechanism, gem-difluorovinyl amide **3j** was treated with Ag_2_CO_3_ in DCM at 100 °C for 12 h, which led to **4v** in 44% yield as detected by ^19^F NMR analysis of the crude reaction mixture [Eq. (4)]. The observation supported our hypothesis that fluorovinylation of amide via the intermediate gem-difluorovinyl amide, so the silver carbonate acted both as a catalyst in the difluorovinylation process and as a base leading to further ring closure.

### Difluoroethylation of amines

**Fig. 7:**
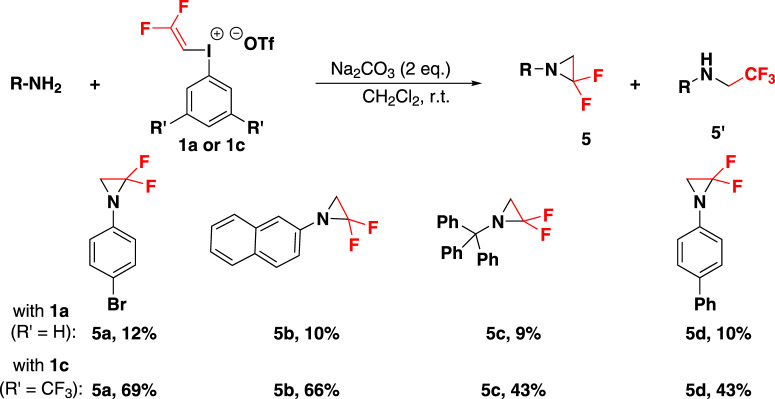
The electrophilic difluoroethylation of amines. Reaction conditions: amine (0.2 mmol), **1** (0.24 mmol), Na_2_CO_3_ (0.4 mmol) in CH_2_Cl_2_ (2.0 ml) at room temperature for 12 h. Yields shown are of isolated products.

Finally, the reaction of DFVI with amines was examined. Initially, *para*-bromoaniline was chosen as the template substrate with **1a** for our optimization studies. However, the target product aziridine **5a** was obtained in only 4% yield, along with 9% of the side product trifluoroethyl amine **5a’** employing Et_3_N base (Fig. 7). It probably arose from the gem-difluorovinylation of amine (α position of alkene reacted with nitrogen atom) subsequently HF generated in the system addition to the double bond. In order to solve the problem of regioselectivity, we tried reagent **1c** with CF_3_ groups on the phenyl ring to make amine attack β-C in priority and indeed the yield of gem-difluoro aziridine was improved to 69%. In addition to this, naphthyl amine was also proved reactive partners, affording the product in moderate yield (**5b**). Both benzyl and biphenyl amine were transformed to generate **5c** and **5d** (43% and 43%) in decreased yield might owing to the instability of difluoro aziridines^58^. However, gem-difluorovinyl ethers and thioethers could not be obtained when combining gem-difluorovinyl iodonium salt with corresponding substrates, and undetermined polymer was detected.

### Proposed mechanism

**Fig. 8:**
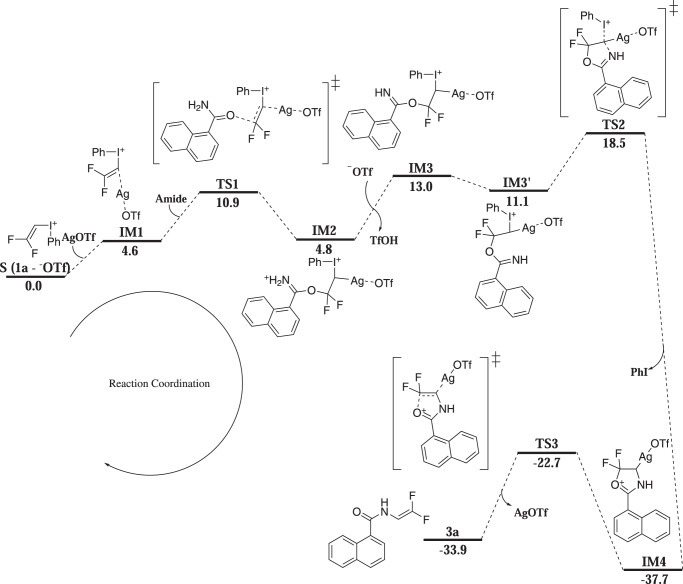
Computed Gibbs free energy profile of the gem-difluorovinylation of amide. The energies are given in kcal/mol.

To gain more insight into the reaction mechanism, density functional theory calculations were conducted. Figure 8 shows the free energy profile of the route leading to the construction of the enamide in a simple model reaction of amide and **1a**. Our computations reveal that the reaction is initiated by a weak interaction between the iodonium cation and the silver salt to form **IM1**. This interaction increases the electrophilicity of the C=CF_2_ bond, and a subsequent Michael Addition of amide to **IM1** renders the formation of **IM2**, proceeding with a low activation barrier of 6.3 kcal/mol. Then, the reaction becomes a little bit endergonic in the deprotonation process using triflate anion. The resulting species **IM3** undergoes five-membered cyclic transition state (**TS2**) when it is transformed into the appropriate conformation **IM3’**. Final product **3a** is obtained upon ring cleavage of **IM4** via **TS3**. This is the rate-determining step with a moderate free energy barrier of 15.0 kcal/mol.

**Fig. 9:**
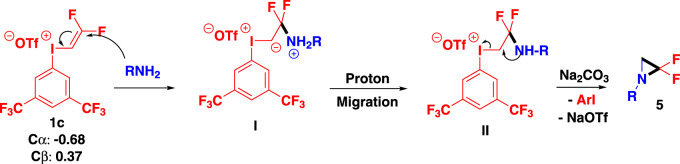
Proposed reaction mechanism for the formation of aziridines iodonium yield. The reaction proceeds via iodonium yield intermediate.

Based on the above results and previous researcher’s work^59^, a plausible reaction mechanism for the ring-closure of amine is proposed in Fig. 9. In which amine attacks the β-C of the alkenyl group to generate the iodonium yield **I** under the basic reaction conditions, subsequently elimination of iodobenzene from **II** upon proton migration from nitrogen to carbon to afford the aziridine salt. Following deprotonation with Na_2_CO_3_, the resultant difluoro aziridine **5** is formed.

## Conclusions

In summary, a bench-stable crystalline of gem-difluorovinyl iodonium salt (DFVI) was developed via the ligand exchange of Stang’s reagent with tributyl(2,2-difluorovinyl)stannane and served as a diverse gem-difluorovinyl reagent. The direct difluoroethylation reactions of N- and O-nucleophiles including amides and acids were realized for the first time with DFVI. Moreover, a number of applications of DFVI were explored, especially in the construction of various fluorinated heterocycles. Considering the unique properties and advantages of this gem-difluorovinyl iodonium salt (DFVI), the reagent might have wider applications in fluorination chemistry and remarkable transformations to investigate.

## Methods

### General procedure for gem-difluorovinylation of carboxylic acid with reagent 1a

Carboxylic acid (0.2 mmol 1.0 equiv.), Ag_2_CO_3_ (4.0 equivalent/1.0 equivalent carboxyl group) and reagent **1a** (4.0 equivalent/1.0 equivalent carboxyl group) were placed into an oven-dried Schlenk tube that is equipped with a stirring bar under N_2_. The tube was quickly sealed with a rubber stopper and 2 ml of freshly distilled CH_3_CN was added. The reaction was stirred at room temperature for 12 h. Then the reaction mixture was concentrated in vacuo and purified by flash chromatography on silica gel.

### General procedure for gem-difluorovinylation of amide with reagent 1a

Amide (0.8 mmol 4.0 equiv.), Ag_2_CO_3_ (10% mol) and reagent **1a** (0.2 mmol 1.0 equiv.) were placed into an oven-dried Schlenk tube that is equipped with a stirring bar under N_2_. The tube was quickly sealed with a rubber stopper and 2 ml of freshly distilled CH_2_Cl_2_ was added. The reaction was stirred at room temperature for 12 h. Then the reaction mixture was concentrated in vacuo and purified by flash chromatography on silica gel.

### General procedure for fluorovinylation of amide with reagent 1a

Amide (0.2 mmol 1.0 equiv.), Ag_2_CO_3_ (0.4 mmol 2.0 equiv.) and reagent **1a** (0.24 mmol 1.2 equiv.) were placed into an oven-dried Schlenk tube that is equipped with a stirring bar under N_2_. The tube was quickly sealed with a rubber stopper and 2 ml of freshly distilled CH_2_Cl_2_ was added. The reaction was stirred at room temperature for 12 h. Then the reaction mixture was concentrated in vacuo and purified by flash chromatography on silica gel.

### General procedure for difluoroethylation of amine with reagent 1c

Amine (0.2 mmol 1.0 equiv.), Na_2_CO_3_ (0.4 mmol 2.0 equiv.) and reagent **1c** (0.24 mmol 1.2 equiv.) were placed into an oven-dried Schlenk tube that is equipped with a stirring bar under N_2_. The tube was quickly sealed with a rubber stopper and 2 ml of freshly distilled CH_2_Cl_2_ was added. The reaction was stirred at room temperature for 12 h. Then the reaction mixture was concentrated in vacuo and purified by flash chromatography on silica gel.

## Supplementary information

Supplementary Information

Description of Additional Supplementary Files

Supplemental Data 1

Supplemental Data 2

Supplemental Data 3

Supplementary Data 4

Supplementary Data 5

## Data Availability

The data supporting the findings of this study are available within the article and its Supplementary Information. Supplementary Methods includes the details of manipulation, isolation and characterization of all new compounds obtained this study. Supplementary Data 4 includes XYZ coordinates for all optimized structures. Supplementary Data 5 includes ^1^H, ^13^C, ^19^F NMR spectra. In addition, the X-ray crystallographic data are included in Supplementary Data 1–3. The X-ray crystallographic coordinates for the structure reported in this article have been deposited at the Cambridge Crystallographic Data Centre (CCDC), under CCDC deposition numbers 2141360 (**1a**), 2141364 (**3a**) and 2141361 (**4j**). These data can be obtained free of charge from the CCDC via http://www.ccdc.cam.ac.uk/data_request/cif. Experimental procedures and characterization of the new compounds are available in the Supplementary Information
