# Supplementary material for: Diverse reactivity of the gem-difluorovinyl iodonium salt for direct incorporation of the difluoroethylene group into N- and O-nucleophiles
Source: Commun Chem. 2022 Dec 3;5:167. doi: 10.1038/s42004-022-00772-7 (PMC9814539; doi:10.1038/s42004-022-00772-7)
Supplement: Supplementary file 1 — Supplementary Information [file 42004_2022_772_MOESM1_ESM.pdf]

Supplementary Information

Diverse reactivity of the gem-difluorovinyl iodonium salt for direct incorporation of the difluoroethylene group into N- and O-nucleophiles

Chenxin Ge[a], Bin Wang[a], Yunchen Jiang[a], Chao Chen[a,b]

a Chenxin Ge, Bin Wang, Yunchen Jiang, Prof. Dr. Chao Chen

Key Laboratory of Bioorganic Phosphorus & Chemical Biology (Ministry of Education, MOE), Tsinghua University, Beijing 100084, China.

E-mail:gcx17@mails.tsinghua.edu.cn;

chenchao01@mails.tsinghua.edu.cn.

b Prof. Dr. Chao Chen

State Key Laboratory of Elemento-Organic Chemistry, Nankai University, Tianjin 300071, China.

Table of contents

Supplementary Methods 3
1. General Information 3
2. Preparation of the starting materials 1a-1c 4
2.1 Preparation of tributyl (2,2-difluorovinyl)stannane 4
2.2 Preparation of (2,2-difluorovinyl)(aryl)-λ^3-iodaneyl trifluoromethanesulfonate 4
3. Optimization of difluorovinylation of carboxylic acid with reagent 1a 6
3.1 Additive 6
3.2 Solvent 7
3.3 Equivalent of starting materials and base 8
4. Optimization of difluorovinylation of amide with reagent 1a 9
4.1 Catalyst 9
4.2 Solvent 10
4.3 Temperature and additive 11
4.4 Equivalent of starting materials 12
5. Optimization of fluorovinylation of amide with reagent 1a 13
5.1 Solvent 13
5.2 Temperature 14
5.3 Base 15
5.4 Silver salt 16
5.5 Equivalent of staring materials 17
6. Optimization of difluoroethylation of amine with reagent 1 18
7. Experimental procedure 19
8. Mechanism study 20
8.1 Reaction between silver benzoate and iodonium salt 20
8.2 Investigation on the interaction of 1a with amide 20
8.3 Study on ring formation of gem-difluorovinyl amide 22
9. X-Ray crystallographic data 23
10. Analytical data 27
11. Quantum chemical calculations 43
11.1 Computational methodology 43
12. Supplementary References 44

Supplementary Methods

1. General Information

^{1}H NMR, ^{13}C NMR and ^{19}F NMR were recorded on (JEOL) JNM-ECS400, JNM-ECZ400S with $CDCl_3$, CD_3CN or DMSO-d_6 as solvent. The chemical shifts are reported in ppm relative to $CDCl_3$ (δ = 7.26), CD_3CN (δ = 1.94), DMSO-d_6 (δ = 2.50) for ^{1}H NMR and relative to the central $CDCl_3$ (δ = 77.16), CD_3CN (δ = 1.32), DMSO-d_6 (δ = 39.52) for ^{13}C NMR. NMR data of known compounds are in agreement with literature values. Coupling constants (*J*) are quoted in Hz. at 400 MHz for ^{1}H. Multiplicities are reported as follows: singlet (s), doublet (d), triplet (t), quartet (q), and multiplet (m). High-resolution mass spectra were acquired on LCMS-IT/TOF (Shimadzu.Japan) in an 80% acetonitrile - 20% water mixture and GCMS (Thermo Fisher Scientific) in DCM.

Materials and Methods: Unless otherwise noted, all reactions were performed under an atmosphere of dry N_2 with oven-dried glassware. Reactions were monitored by analytical thin layer chromatography (TLC) on 0.20-0.30 mm Yantai Chemical Industry silica gel plates and spots were detected by 254 nm UV-absorption. Silica gel (200-300 mesh) (from Qingdao Marine Chem. Company, Ltd.) was used for flash chromatography. Substrates were synthesized and purified according to the literature procedures. Other chemicals or reagents were obtained from commercial sources.

2. Preparation of the starting materials 1a-1c

2.1 Preparation of tributyl (2,2-difluorovinyl)stannane

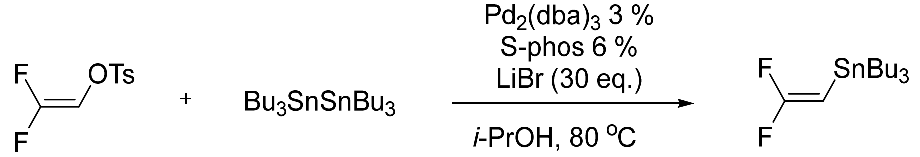

A suspension of 2,2-difluorovinyl 4-methylbenzenesulfonate (10 mmol), $Pd_2(dba)_2$ (0.3 mmol), S-phos (0.6 mmol), LiBr (300 mmol) in 50 mL of propan-2-ol was degassed three times with freeze/pump/thaw technique in a flame-dried round bottom flask. 1.0 Equiv. of 1,1,1,2,2,2-hexabutyldistannane (10 mmol) was added using a syringe to the thawed solution under nitrogen. The reaction was allowed to react at 80 °C for 12 hours and solvent was removed in vacuo. Then 50 ml of EA was added and the organic layer was washed with a saturated solution of potassium fluoride. The precipitate was filtered through celite and washed with EA (2 × 30 mL). Then organic layer was dried over anhydrous Na_2SO_4. Solvent was removed in vacuo. The reaction mixture was purified by flash chromatography on silica gel (eluent: PE) to afford crude product. Pure product was obtained when crude product was distilled under high vacuum (b.p.: 46 ° C (0.4 Pa)), the spectroscopy was consistent with those reported in the literature.[1]

2.2 Preparation of (2,2-difluorovinyl)(aryl)-λ^3-iodaneyl trifluoromethanesulfonate

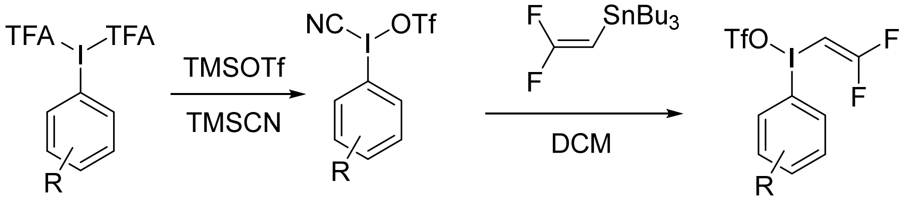

6a- 6c

1a - 1c

Trimethylsilyltrifluoromethanesulfonate (1.4 mL, 7.8 mmol) was added to a stirred solution of bis(trifluoroacetoxy)iodo benzene (3.1 g, 7.2 mmol) in CH_2Cl_2 (25 mL) at -20 °C. The mixture was warmed to ambient temperature for 10 min until the color of the solution changed from colorless to yellow. The reaction mixture was re-cooled to -30 °C, and trimethylsilyl cyanide (1.0 mL, 7.8 mmol) was added via syringe resulting in the formation of a yellow-white salt 6a. Et_2O was added to the reaction mixture and solvent was removed through catheter tied with filter paper below 0 °C. Wash the solid twice with ether to afford pure 6a. 6b-6c was synthesized according to the reported literature.[2]

In a Schlenk tube (100 mL), a portion of the above salt (6 mmol) was suspended in

CH_2Cl_2 (40 mL) and cooled to -40 °C. Tributyl(vinyl)tin (6 mmol) dissolved in CH_2Cl_2 (10 mL) was then added dropwise to the stirring solution. After the addition was complete, the cooling bath was removed and the reaction allowed to warm to ambient temperature. After 2 hours the slightly yellow solution was concentrated in vacuo leaving approx. 5 mL CH_2Cl_2 to which Et_2O (20 mL) was added with vigorous stirring and -20 °C. The precipitate was filtered and washed with Et_2O (3×10 mL), 1a - 1c was obtained as a white solid (1a: 68% yield, 1b: 58 % yield, 1c: 65 %yield.).

(2,2-difluorovinyl)(phenyl)-λ^3-iodanyl trifluoromethanesulfonate (1a)

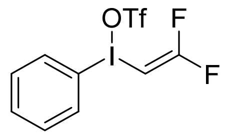

White solid 1.70 g (4.08 mmol, 68%). **^{1}H** NMR (400 MHz, DMSO-d_6) δ 8.18 (d, J = 8.3 Hz, 2H), 7.72 (t, J = 7.3 Hz, 1H), 7.58 (t, J = 7.8 Hz, 2H), 6.97 (dd, J = 26.6, 1.8 Hz, 1H). **^{13}C NMR** (101 MHz, DMSO- d_6) δ 158.6 (dd, J = 304.9, 292.4 Hz), 135.2, 132.3, 131.9, 120.7 (q, J = 321.7 Hz, OTf), 115.8, 57.5 (dd, J = 33.7, 21.2 Hz). **^{19}F NMR** (376 MHz, DMSO- d_6) δ -65.02 (dd, J = 26.0, 6.5 Hz, 1F), -67.56 (d, J = 8.7 Hz, 1F), -77.68 (s, 3F). **HRMS (ESI-TOF)** calculated for $C_8H_6F_2I$ [M-OTf]$^+$ 266.9482; found: 266.9470.

(2,2-difluorovinyl)(4-(trifluoromethyl)phenyl)-λ^3-iodanyl trifluoromethanesulfonate (1b)

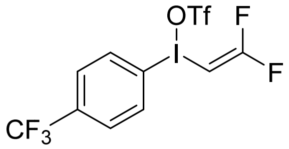

White solid 1.69 g (3.48 mmol, 58%). **^{1}H NMR** (400 MHz, Acetonitrile-d_3) δ 8.27 (d, J = 8.7 Hz, 2H), 7.86 (d, J = 8.7 Hz, 2H), 6.42 (d, J = 24.3 Hz, 1H). **^{13}C NMR** (101 MHz, Acetonitrile-d_3) δ 161.5 (dd, J = 307.8, 295.2 Hz), 137.3, 134.8 (q, J = 33.7 Hz), 129.7 (q, J = 4.8 Hz), 124.4 (q, J = 272.6 Hz, CF_3), 121.7 (q, J = 320.6 Hz, OTf), 118.0, 55.7 (dd, J = 41.4, 22.2 Hz). **^{19}F NMR** (376 MHz, Acetonitrile-d_3) δ -61.67 (d, J = 23.8 Hz, 1F), -63.77 (s, 3F), -64.07 (s, 1F), -79.27 (s, 3F). **HRMS (ESI-TOF)** calculated for $C_9H_5F_5I$ [M-OTf]$^+$ 334.9356; found: 334.9347.

(3,5-bis(trifluoromethyl)phenyl)(2,2-difluorovinyl)-λ^3-iodanyl trifluoromethanesulfonate (1c)

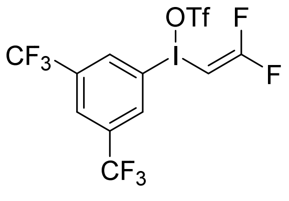

White solid 2.15 g (3.9 mmol, 65%). **^{1}H** NMR (400 MHz, Acetonitrile-d_3) δ 8.68 (s, 2H), 8.34 (s, 1H), 6.46 (d, J = 24.3 Hz, 1H). **^{13}C NMR** (101 MHz, Acetonitrile-d_3) δ

161.6 (dd, J = 307.8, 295.2 Hz), 137.4 (d, J = 3.9 Hz), 134.5 (q, J = 34.7 Hz), 128.2, 123.2 (q, J = 273.0 Hz, CF_3), 121.5 (q, J = 319.5 Hz, OTf), 114.2, 56.2 (dd, J = 40.9, 22.6 Hz). **^{19}F NMR** (376 MHz, Acetonitrile-d_3) δ -60.47 (dd, J = 24.9, 5.2 Hz, 1F), -63.42 (s, 6F), -63.57 (d, J = 4.3 Hz, 1F), -79.38 (s, 3F). **HRMS (ESI-TOF)** calculated for $C_{10}H_4F_8I$ $[M\text{-}OTf]^+$ 402.9230; found: 402.9228.

3. Optimization of difluorovinylation of carboxylic acid with reagent 1a

3.1 Additive

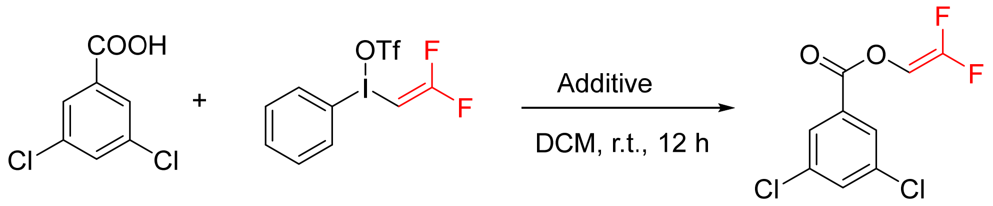

0.05 mmol **1a** (0.05 mmol)

Entry[a]	Additive	Equiv.	Yield (%)[b]
1	—		0
2	CuBr	0.1	trace
3	Cu_2O	2	0
4	Ag_2CO_3	0.1	3
**5**	**Ag_2CO_3**	**2**	**10**

[a]Reaction conditions: 3,5-dichlorobenzoic acid (0.05 mmol), **1a** (0.05 mmol), additive in DCM (0.5 mL) at 25 °C for 12 h; [b]Yields were determined by ^{19}F NMR analysis of the crude reaction mixture with an internal standard *p*-fluoroiodobenzene.

3.2 Solvent

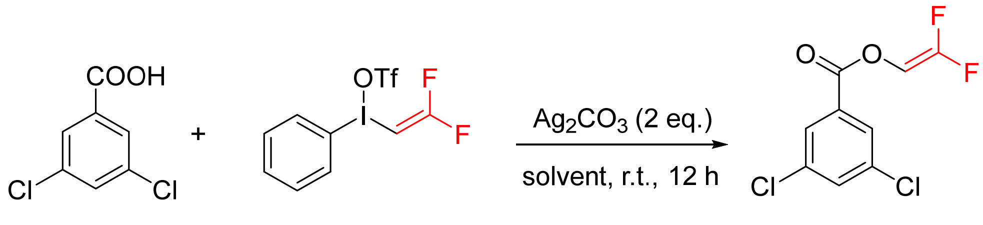

Entry[a]	solvent	Yield (%)[b]
1	THF	8
2	Et_2O	1
3	DCE	17
**4**	**CH_3CN**	**59**
5	Toluene	15

[a]Reaction conditions: 3,5-dichlorobenzoic acid (0.05 mmol), **1a** (0.05 mmol), Ag_2CO_3 (0.1 mmol) in solvent (0.5 mL) at 25 °C for 12 h; [b]Yields were determined by ^{19}F NMR analysis of the crude reaction mixture with an internal standard *p*-fluoroiodobenzene.

3.3 Equivalent of starting materials and base

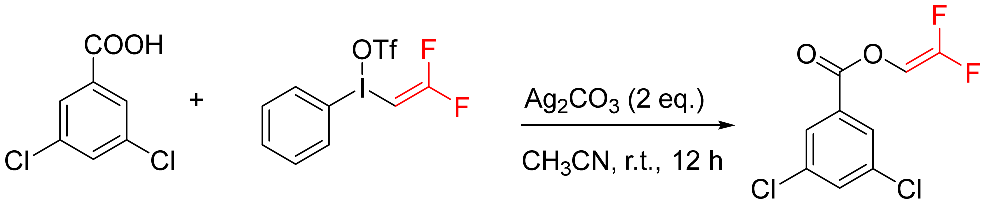

1a

Entry[a]	Equiv. of acid	Equiv. of **1a**	Yield (%) [b,c]
1	2	1	51
2	4	1	49
3	1	2	64
4	1	4	71
**5**[d]	**1**	**4**	**80 (76)**

[a]Reaction conditions: 3,5-dichlorobenzoic acid, **1a**, Ag_2CO_3 (0.1 mmol) in CH_3CN (0.5 mL) at 25 °C for 12 h; [b]Yields were determined by ^{19}F NMR analysis of the crude reaction mixture with an internal standard *p*-fluoroiodobenzene. [c]isolated yield in parentheses; [d]4 equivalent Ag_2CO_3 was used.

4. Optimization of difluorovinylation of amide with reagent 1a

4.1 Catalyst

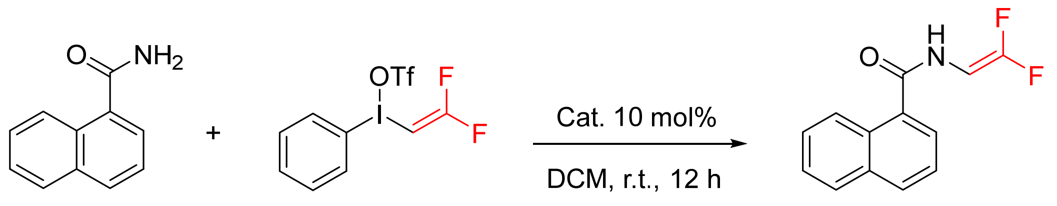

Entry[a]	cat.	Yield (%)[b]
**1**	**Ag_2CO_3**	**36**
2	AgOTf	21
3	$AgNO_3$	trace
4	AgOAc	20
5	Ag_2O	23

[a]Reaction conditions: 1-naphthamide (0.05 mmol), **1a** (0.05 mmol), cat. (0.005 mmol) in DCM (0.5 mL) at 25 °C for 12 h; [b]Yields were determined by ^{19}F NMR analysis of the crude reaction mixture with an internal standard *p*-fluoroiodobenzene.

4.2 Solvent

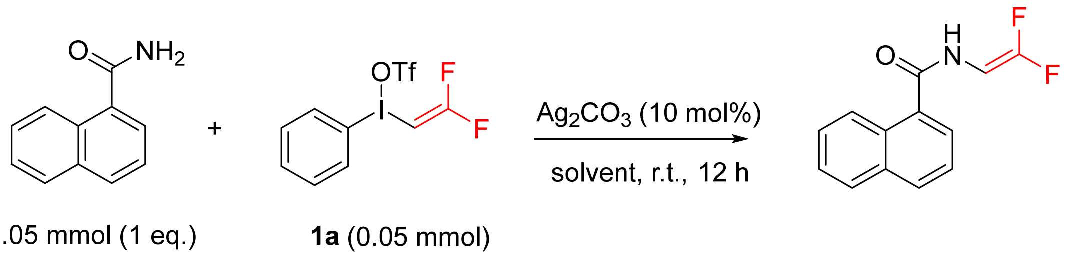

Entry[a]	solvent	Yield (%)[b]
1	THF	0
2	DCE	24
3	CH_3CN	0
4	Toluene	0
5	Et_2O	0
6	$CHCl_3$	0

[a]Reaction conditions: 1-naphthamide (0.05 mmol), **1a** (0.05 mmol), Ag_2CO_3 (0.005 mmol) in solvent (0.5 mL) at 25 °C for 12 h; [b]Yields were determined by ^{19}F NMR analysis of the crude reaction mixture with an internal standard *p*-fluoroiodobenzene.

4.3 Temperature and additive

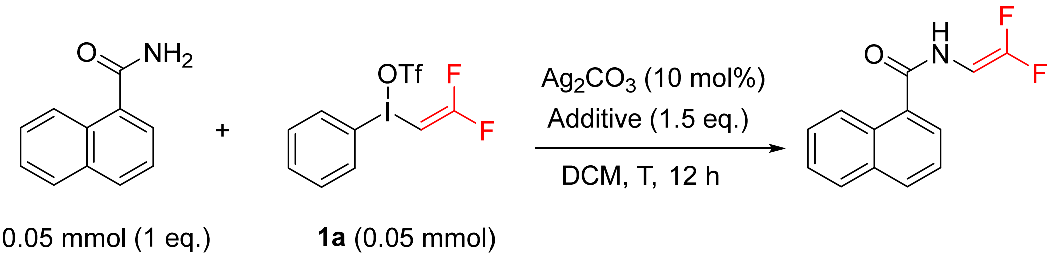

Entry[a]	T	Additive	Yield (%)[b]
1	40	—	24
2	60	—	17
3	80	—	9
4	0	—	0
5	-20	—	0
6	25	LiOTf	15
7	25	NaOTf	28

[a]Reaction conditions: 1-naphthamide (0.05 mmol), **1a** (0.05 mmol), Ag_2CO_3 (0.005 mmol), Additive (0.075 mmol) in DCM (0.5 mL) at T °C for 12 h; [b]Yields were determined by ^{19}F NMR analysis of the crude reaction mixture with an internal standard *p*-fluoroiodobenzene.

4.4 Equivalent of starting materials

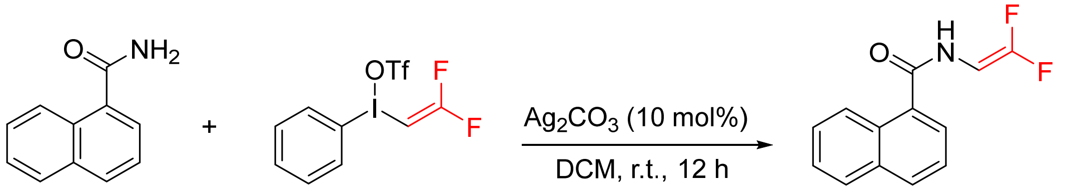

x eq. **1a** (0.05 mmol)

Entry[a]	X	Yield (%)[b,c]
1	2	68
2	3	74
**3**	**4**	**85 (82)**

[a]Reaction conditions: 1-naphthamide, **1a** (0.05 mmol), Ag_2CO_3 (0.005 mmol) in DCM (0.5 mL) at 25 °C for 12 h; [b]Yields were determined by ^{19}F NMR analysis of the crude reaction mixture with an internal standard *p*-fluoroiodobenzene; [c]isolated yield in parentheses

5. Optimization of fluorovinylation of amide with reagent 1a

5.1 Solvent

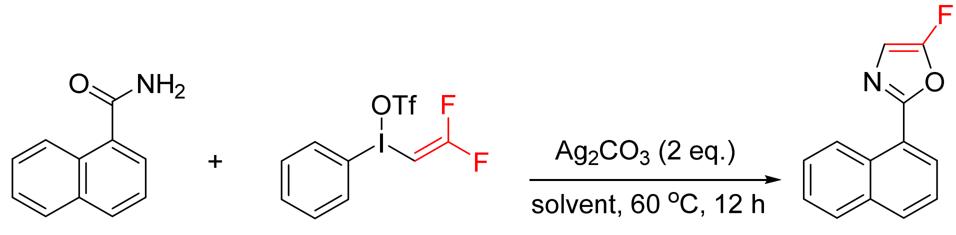

Entry[a]	solvent	Yield (%)[b]
1	^{t}BuOMe	14
2	CH_3CN	0
3	DCE	30
4	THF	3
**5**	**DCM**	**49**
6	toluene	16
7	Et_2O	17
8	EtOAc	23
9	DMF	0
10	MeOH	0
11	HFIP	0

[a]Reaction conditions: 1-naphthamide (0.05 mmol), **1a** (0.05 mmol), Ag_2CO_3 (0.10 mmol) in solvent (0.5 mL) at 60 °C for 12 h; [b]Yields were determined by ^{19}F NMR analysis of the crude reaction mixture with an internal standard *p*-fluoroiodobenzene.

5.2 Temperature

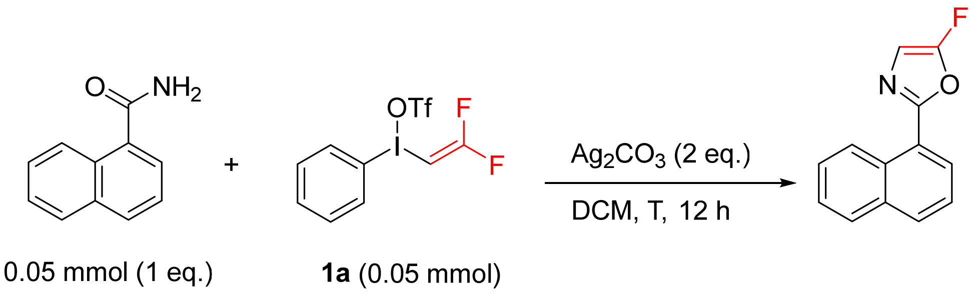

Entry[a]	T/°C	Yield (%)[b]
**1**	**25**	**54**
2	40	51
3	50	47
4	70	46
5	80	38

[a]Reaction conditions: 1-naphthamide (0.05 mmol), **1a** (0.05 mmol), Ag_2CO_3 (0.10 mmol) in DCM (0.5 mL) at T °C for 12 h; [b]Yields were determined by ^{19}F NMR analysis of the crude reaction mixture with an internal standard *p*-fluoroiodobenzene.

5.3 Base

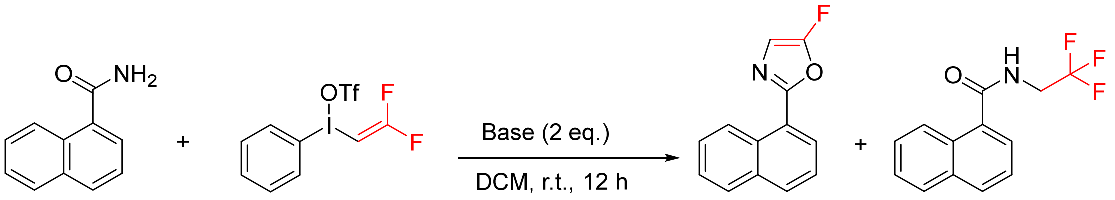

0.05 mmol (1 eq.)   **1a** (0.05 mmol)   **4a**   **4a'**

Entry[a]	Base	Yield (%)[b]	
		**4a**	**4a'**
1	K_2CO_3	0	28
2	K_3PO_4	0	8
3	Li_2CO_3	0	0
4	KO^{t}Bu	0	0
5	Na_2CO_3	0	22
6	NaOH	0	8
7[c]	K_2CO_3	0	17
8[c]	Li_2CO_3	0	0
9[c]	Na_2CO_3	0	26
10[c]	$NaHCO_3$	0	trace
11[c]	1-methylindole	0	0
12[c]	DTBPy	0	0

[a]Reaction conditions: 1-naphthamide (0.05), **1a** (0.05 mmol), Base (0.10 mmol) in DCM (0.5 mL) at 25 °C for 12 h; [b]Yields were determined by ^{19}F NMR analysis of the crude reaction mixture with an internal standard *p*-fluoroiodobenzene; [c]Ag_2CO_3 (10 mol%) was added.

5.4 Silver salt

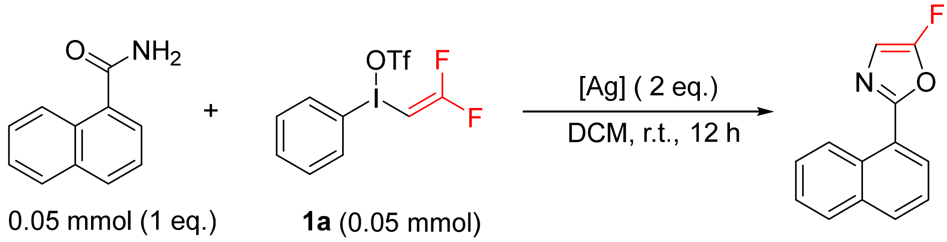

Entry[a]	[Ag]	Yield (%)[b]
**1**	**Ag_2CO_3**	**54**
2[c]	Ag_2CO_3	trace
3[d]	Ag_2CO_3	28
4[e]	Ag_2CO_3	0
5	$AgBF_4$	0
6	AgF	5
7	$AgNO_2$	0
8	AgOAc	28
9	Ag_2O	9

[a]Reaction conditions: 1-naphthamide (0.05 mmol), **1a** (0.05 mmol), [Ag] (0.10 mmol) in DCM (0.5 mL) at 25 °C for 12 h; [b]Yields were determined by ^{19}F NMR analysis of the crude reaction mixture with an internal standard *p*-fluoroiodobenzene; [c]LiOTf (0.1 mmol) was added; [d]NaOTf (0.10 mmol) was added; [e]50 mg 4Å molecular sieve was added.

5.5 Equivalent of staring materials

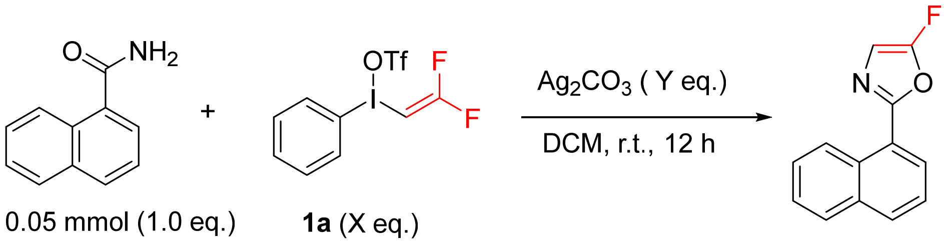

Entry[a]	X	Y	Yield (%)[b,c]
**1**	**1.2**	**2**	**72(69)**
2	1.2	4	24
3	1.2	6	trace
4[d]	1.2	2	68
5[e]	1.2	2	0

[a]Reaction conditions: 1-naphthamide (0.05 mmol), **1a**, Ag_2CO_3 in DCM (0.5 mL) at 25 °C for 12 h; [b]Yields were determined by ^{19}F NMR analysis of the crude reaction mixture with an internal standard *p*-fluoroiodobenzene; [c]isolated yield in parentheses; [d]CuO (0.10 mmol) was added; [e]PIDA (0.10 mmol) was added.

6. Optimization of difluoroethylation of amine with reagent 1

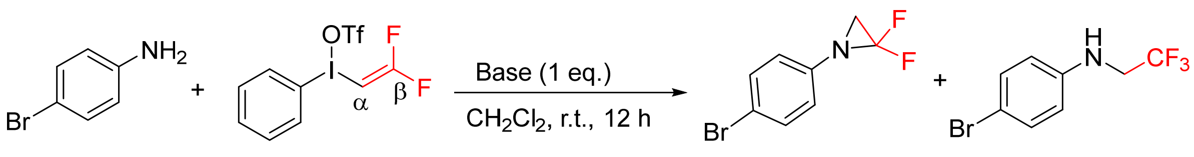

0.05 mmol (1 eq.) **1a** (0.05 mmol) **5a** **5a'**

Entry[a]	Base	Yield (%)[b,c]	
		**5a**	**5a'**
1	Cs_2CO_3	7	0
2	$NaHCO_3$	4	0
3	Et_3N	4	9
4	Na_2CO_3	10	0
5	K_2CO_3	8	0
6[d]	Na_2CO_3	9	0
7[e]	Na_2CO_3	56	0
**8[f]**	$\mathbf{Na_2CO_3}$	**70 (69)**	**0**

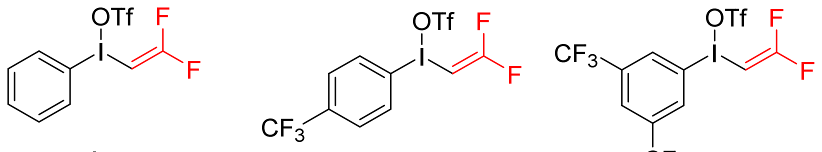

1a **1b** **1c**

[a]Reaction conditions: *p*-bromoaniline (0.05 mmol), **1a** (0.05 mmol), Base (0.10 mmol) in DCM (0.5 mL) at 25 °C for 12 h; [b]Yields were determined by ^{19}F NMR analysis of the crude reaction mixture with an internal standard *p*-fluoroiodobenzene; [c]isolated yield in parentheses; [d]**1a** was replaced with **1b**; [e]**1a** was replaced with **1c**; [f]**1a** was replaced with **1c** (1.2 eq., 0.06 mmol).

7. Experimental procedure

7.1 General procedure for gem-difluorovinylation of carboxylic acid with reagent 1a

Carboxylic acid (0.2 mmol 1.0 equiv.), Ag_2CO_3 (4.0 equivalent / 1.0 equivalent carboxyl group) and reagent **1a** (4.0 equivalent / 1.0 equivalent carboxyl group) were placed into an oven-dried Schlenk tube that is equipped with a stirring bar under N_2. The tube was quickly sealed with a rubber stopper and 2 mL of freshly distilled CH_3CN was added. The reaction was stirred at room temperature for 12 h. Then the reaction mixture was concentrated in vacuo and purified by flash chromatography on silica gel.

7.2 General procedure for gem-difluorovinylation of amide with reagent 1a

Amide (0.8 mmol 4.0 equiv.), Ag_2CO_3 (10% mol) and reagent **1a** (0.2 mmol 1.0 equiv.) were placed into an oven-dried Schlenk tube that is equipped with a stirring bar under N_2. The tube was quickly sealed with a rubber stopper and 2 mL of freshly distilled CH_2Cl_2 was added. The reaction was stirred at room temperature for 12 h. Then the reaction mixture was concentrated in vacuo and purified by flash chromatography on silica gel.

7.3 General procedure for fluorovinylation of amide with reagent 1a

Amide (0.2 mmol 1.0 equiv.), Ag_2CO_3 (0.4 mmol 2.0 equiv.) and reagent **1a** (0.24 mmol 1.2 equiv.) were placed into an oven-dried Schlenk tube that is equipped with a stirring bar under N_2. The tube was quickly sealed with a rubber stopper and 2 mL of freshly distilled CH_2Cl_2 was added. The reaction was stirred at room temperature for 12 h. Then the reaction mixture was concentrated in vacuo and purified by flash chromatography on silica gel.

7.4 General procedure for difluoroethylation of amine with reagent 1c

Amine (0.2 mmol 1.0 equiv.), Na_2CO_3 (0.4 mmol 2.0 equiv.) and reagent **1c** (0.24 mmol 1.2 equiv.) were placed into an oven-dried Schlenk tube that is equipped with a stirring bar under N_2. The tube was quickly sealed with a rubber stopper and 2 mL of freshly distilled CH_2Cl_2 was added. The reaction was stirred at room temperature for 12 h. Then the reaction mixture was concentrated in vacuo and purified by flash chromatography on silica gel.

8. Mechanism study

8.1 Reaction between silver benzoate and iodonium salt

Silver benzoate (0.05 mmol, 1.0 eq.), reagent **1a** (0.05 mmol, 1.0 eq.) were placed into an oven-dried Schlenk tube that is equipped with a stirring bar under N_2. The tube was quickly sealed with a rubber stopper and 0.5 mL of freshly distilled CH_3CN was added. The reaction was stirred at room temperature for 12 h. Then the reaction mixture was added internal standard *p*-fluoroiodobenzene (4 μl) to calculate the NMR yield. Yield 63% (*p*-fluoroiodobenzene ^{19}FNMR (-115.14 - -115.19))

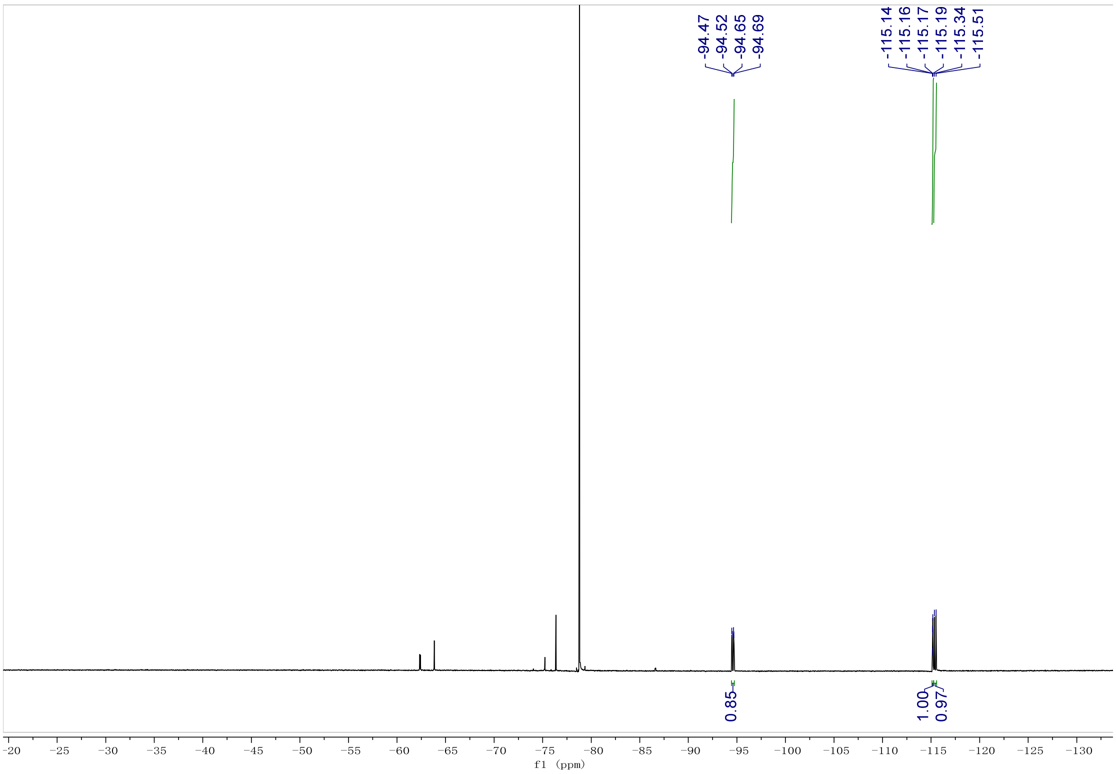

8.2 Investigation on the interaction of 1a with amide

1-naphthamide (0.05 mmol), reagent **1a** (0.05 mmol) were placed into an NMR tube in CD_2Cl_2, comparing the NMR of mixture with starting materials. A significant upfield shift (0.19 ppm) of the F resonance in iodonium salt **1a** and simultaneously a downfield shift (0.34 ppm) of the active N proton signal in amide was observed.

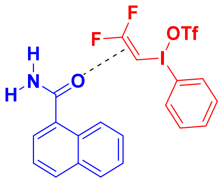

^{19}F NMR of mixture and iodonium salt

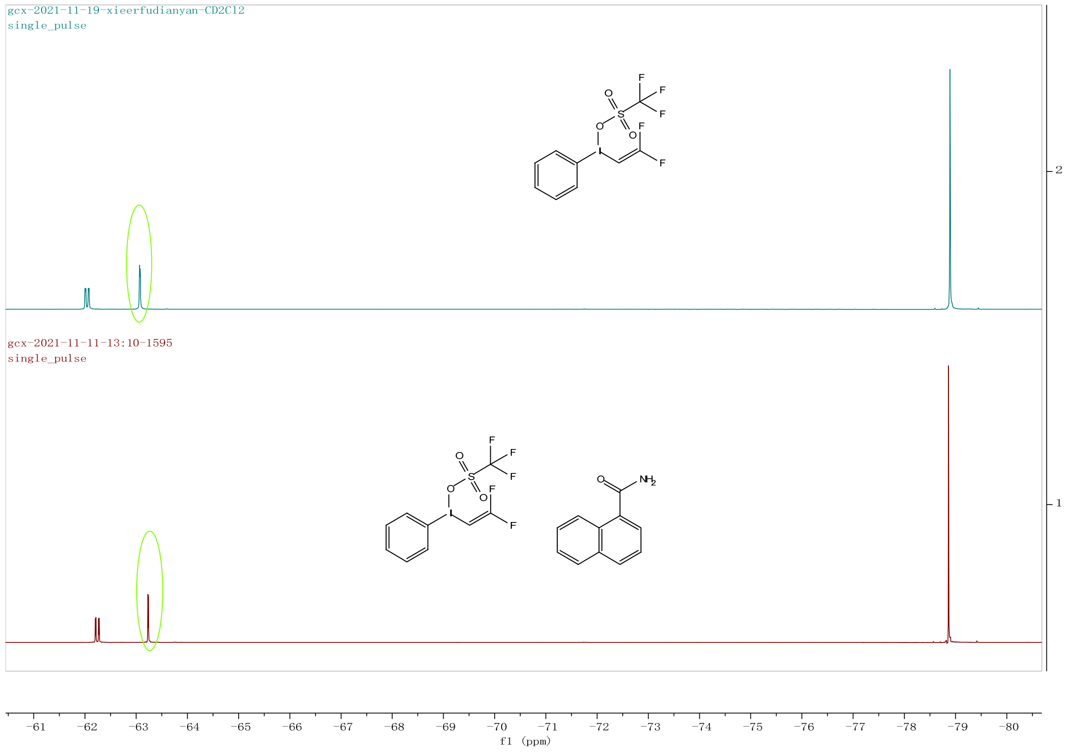

^{1}H NMR of mixture and amide

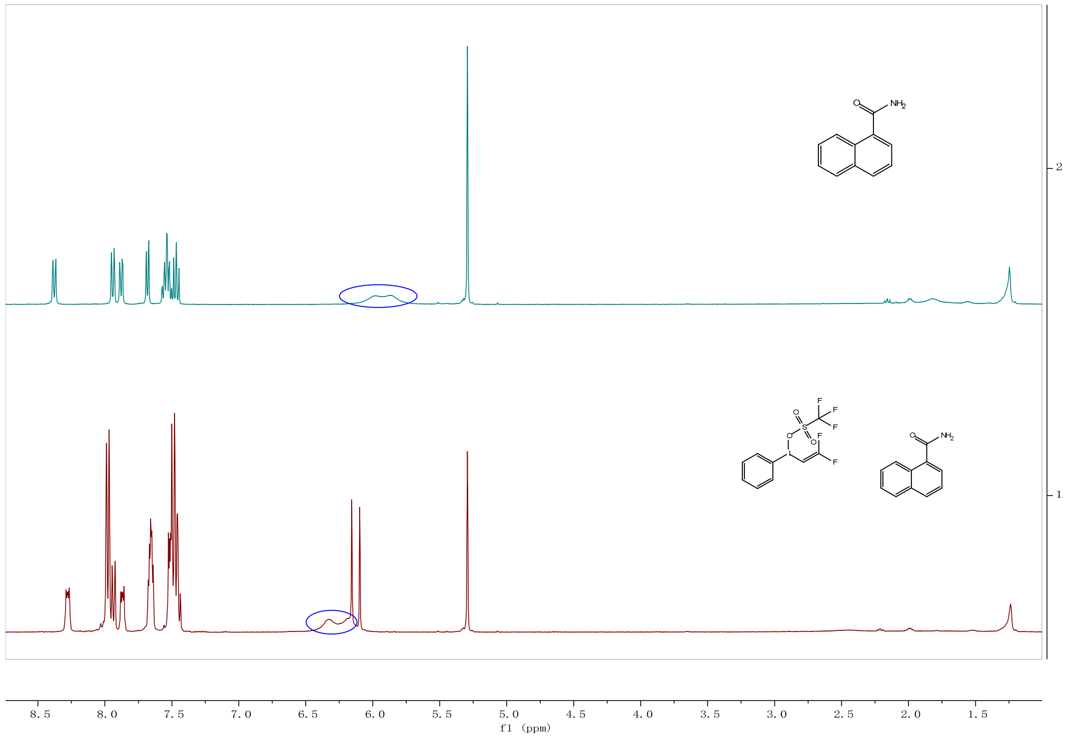

8.3 Study on ring formation of gem-difluorovinyl amide

N-(2,2-difluorovinyl)-2-(naphthalen-1-yl) acetamide (0.065 mmol 1.0 equiv.), Ag_2CO_3 (0.13 mmol, 2.0 equiv.) were placed into an oven-dried Schlenk tube that was equipped with a stirring bar under N_2. The tube was quickly sealed with a rubber stopper and 1 mL of freshly distilled DCM was added. The reaction was stirred at 100 °C for 12 h. Followed by subsequent ^{19}F NMR of reaction mixture. Approximately 44% of N-(2,2-difluorovinyl)-2-(naphthalen-1-yl) acetamide was converted to the title compound 5-fluoro-2-(naphthalen-1-ylmethyl) oxazole.

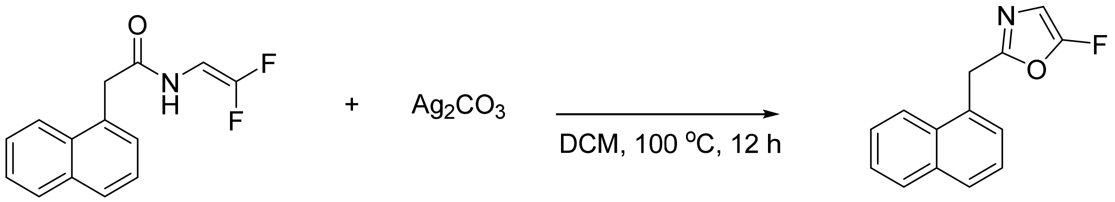

0.065 mmol (1 eq.) 0.13 mmol (2 eq.) **4v**, 44%

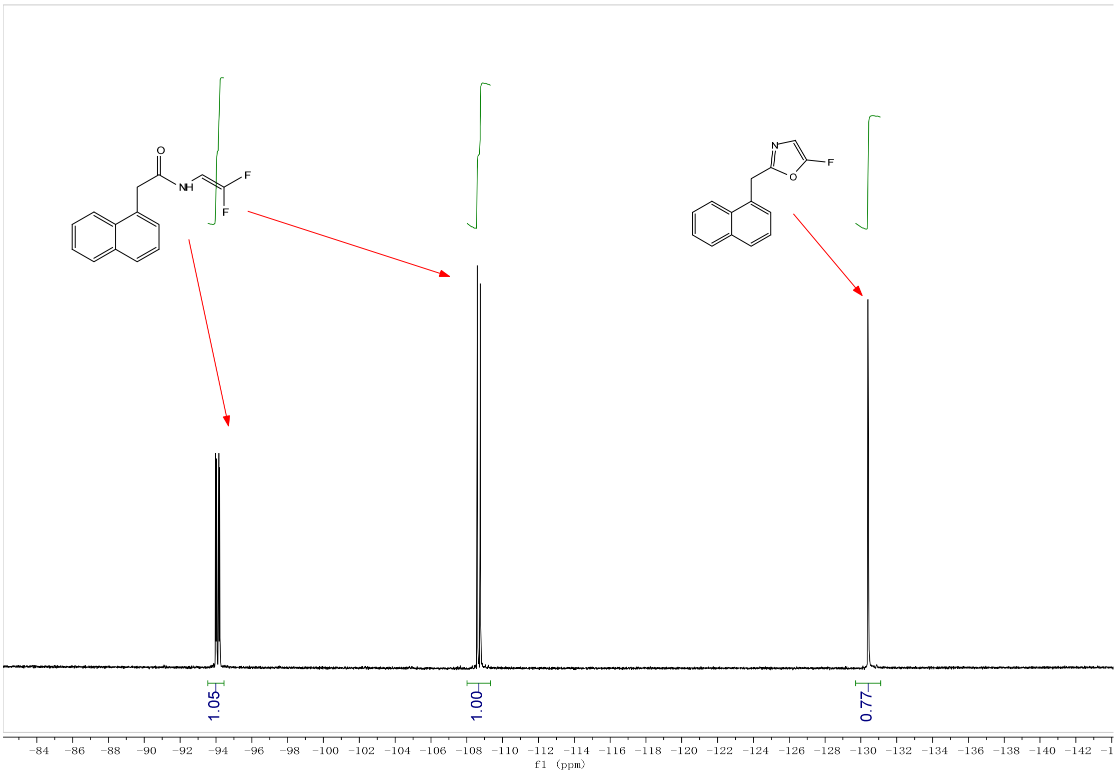

9. X-Ray crystallographic data

The single crystal for compound **1a**, **3a**, **4j** were prepared from a mixture solvent of $CHCl_3$ and diethyl ether or pentane. The data were collected on a Rigaku Oxford Diffract instrument using Cu-Kα radiation (λ = 1.54178 Å) or Mo-Kα (λ = 0.71073 Å) at 173 K or 293 K. Data collection was controlled by CrysAlis^{Pro} (Rigaku,2016). Computations were performed using the SHELXTL NT ver. 5.10 program package (Bruker, 1997) on an IBM PC 586 computer. All non-hydrogen atoms were subjected to anisotropic refinement. The hydrogen atoms were generated geometrically with C-H bonds of 0.93-0.97 Å according to criteria described in the SHELXTL manual (Bruker, 1997). The crystallographic data have already been deposited at the Cambridge Crystallographic Data Centre. CCDC numbers: 2141360 (**1a**), 2141361 (**4j**), 2141364 (**3a**).

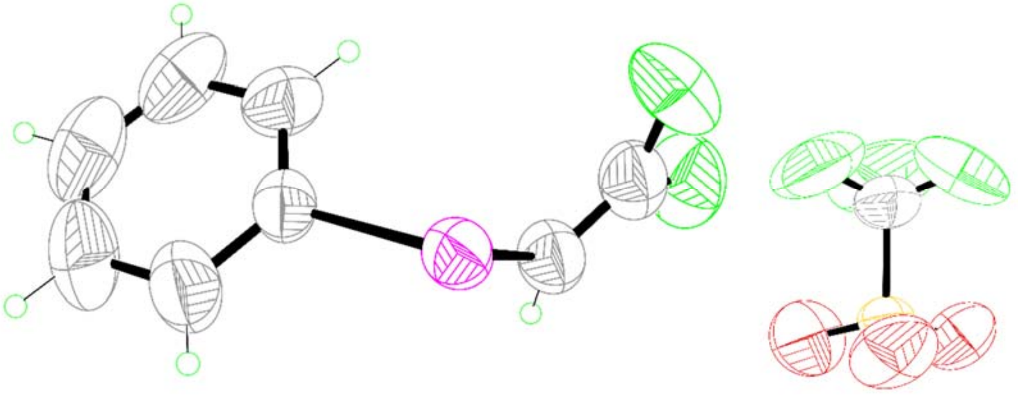

X-ray structure of **1a** (CCDC 2141360)

Table S1. Details of Data Collection, Processing and Structure Refinement

Sample code	**ChenC-42**		
Molecular formula	$[C_8H_6F_2I][CF_3O_3S]$		
Molecular weight	416.10		
Color and habit	colorless block		
Crystal size	0.08 × 0.25 × 0.4 mm		
Crystal system	monoclinic		
Space group	*I*2/*a* (No. 15)		
Unit cell parameters	*a* = 15.7471(10) Å	α = 90.00°	
	b = 8.4612(5) Å	β = 95.039(6)°	
	c = 20.3914(15) Å	γ = 90.00°	
	V = 2706.4(3) Å^3	*Z* = 8	*F*(000) = 1584
Density (calcd)	2.042 g/cm^3		

Diffractometer	SuperNova, Dual, Cu at home/near, AtlasS2
Radiation	Cu K_{α}, λ = 1.54178 Å
Temperature	293±2K
Scan type	ω-scan
Data collection range	$-19 < h < 18$, $-10 < k < 6$, $-23 < l < 24$; θ_{max} = 70.7°
Reflections measured	Total: 7203 Unique (n): 2523 Observed [I ≥ 2σ(I)]: 1913
Absorption coefficient	20.657 mm^{-1}
Minimum and maximum transmission	0.121, 1.000
No. of variables, p	173

Weighting scheme $w = \dfrac{1}{\sigma^2(F_o^2) + (0.0774P)^2 + 8.7651P}$ $P = (F_o^2 + 2F_c^2)/3$

$R1 = \dfrac{\Sigma||F_o|-|F_c||}{\Sigma|F_o|}$ (for all reflections) 0.0636 0.0508 (for observed data)

$wR2 = \sqrt{\dfrac{\Sigma[w(F_o^2 - F_c^2)^2]}{\Sigma w(F_o^2)^2}}$ (for all reflections) 0.1572 0.1431 (for observed data)

Goof = S = $\sqrt{\dfrac{\Sigma[w(F_o^2 - F_c^2)^2]}{n - p}}$ 1.098

Largest and mean Δ/σ 0.001, 0.000

Residual extrema in final difference map -0.984 to 1.219 e $Å^{-3}$

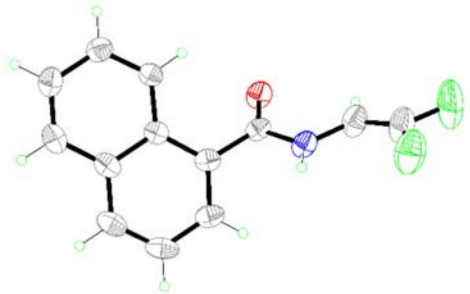

X-ray structure of **3a** (CCDC 2141364)

Table S2. Details of Data Collection, Processing and Structure Refinement

Sample code	**ChenC-40**
Molecular formula	$C_{13}H_9F_2NO$
Molecular weight	233.21
Color and habit	colorless block

Crystal size	0.3 × 0.4 × 0.45 mm
Crystal system	monoclinic
Space group	$P2_1/c$ (No. 14)
Unit cell parameters	a = 21.0063(3) Å α = 90.00°
	b = 5.50650(10) Å β = 98.215(2)°
	c = 9.7623(2) Å γ = 90.00°
	V = 1117.63(3) Å^3 Z = 4 F(000) = 480
Density (calcd)	1.386 g/cm^3
Diffractometer	XtaLAB AFC11 (RINC): quarter-chi single
Radiation	Cu K_α, λ = 1.54178 Å
Temperature	178±2K
Scan type	ω-scan
Data collection range	-24 < h < 24, -6 < k < 6, -8 < l < 11; θ_{max} = 66.5°
Reflections measured	Total: 7201 Unique (n): 1871 Observed [I ≥ 2σ(I)]: 1810
Absorption coefficient	0.948 mm^{-1}
Minimum and maximum transmission	0.717, 1.000
No. of variables, p	158

Weighting scheme $$w = \frac{1}{\sigma^2(F_o^2) + (0.0404P)^2 + 0.2492P} \qquad P = (F_o^2 + 2F_c^2)/3$$

$R1 = \dfrac{\Sigma||F_o|-|F_c||}{\Sigma|F_o|}$ (for all reflections) 0.0348 0.0340 (for observed data)

$wR2 = \sqrt{\dfrac{\Sigma[w(F_o^2 - F_c^2)^2]}{\Sigma w(F_o^2)^2}}$ (for all reflections) 0.0891 0.0886 (for observed data)

Goof = S = $\sqrt{\dfrac{\Sigma[w(F_o^2 - F_c^2)^2]}{n - p}}$ 1.097

Largest and mean Δ/σ 0.001, 0.000

Residual extrema in final difference map -0.130 to 0.139 e Å^{-3}

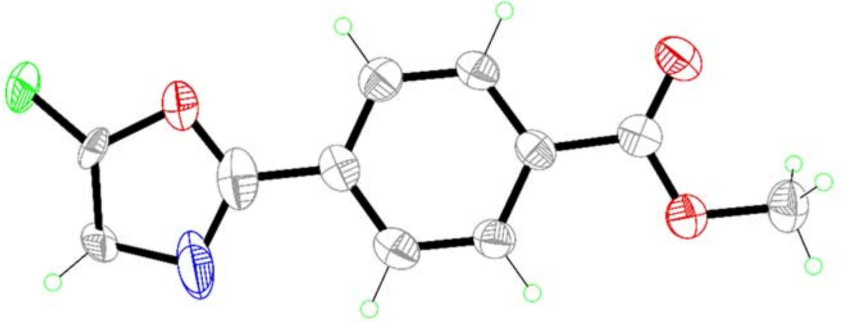

X-ray structure of **4j** (CCDC 2141361)

Table S3. Details of Data Collection, Processing and Structure Refinement

Sample code	**ChenC-32**
Molecular formula	$C_{11}H_8FNO_3$
Molecular weight	221.18
Color and habit	colorless plate
Crystal size	0.05 × 0.15 × 0.30 mm
Crystal system	monoclinic
Space group	$P2_1/c$ (No. 14)
Unit cell parameters	a = 3.9432(4) Å α = 90.00°
	b = 6.0596(7) Å β = 91.270(10)°
	c = 40.388(5) Å γ = 90.00°
	V = 964.80(18) Å^3 Z = 4 F(000) = 456
Density (calcd)	1.523 g/cm^3
Diffractometer	SuperNova, Dual, Cu at zero, AtlasS2
Radiation	Mo K$_\alpha$, λ = 0.71073 Å
Temperature	173(2)°K
Scan type	ω-scan
Data collection range	$-5 < h < 5$, $-8 < k < 7$, $-50 < l < 48$; θ_{max} = 29.5°
Reflections measured	Total: 4694 Unique (n): 2280 Observed [I ≥ 2σ(I)]: 1156
Absorption coefficient	0.124 mm^{-1}
Minimum and maximum transmission	0.900, 1.000
No. of variables, p	191
Weighting scheme	$w = \dfrac{1}{\sigma^2(F_o^2) + (0.065P)^2}$ $P = (F_o^2 + 2F_c^2)/3$
$R1 = \dfrac{\Sigma\|\|F_o\|-\|F_c\|\|}{\Sigma\|F_o\|}$ (for all reflections)	0.1389 0.0636 (for observed data)

$wR2 = \sqrt{\frac{\Sigma[w(F_o^2 - F_c^2)^2]}{\Sigma w(F_o^2)^2}}$ (for all reflections) 0.1752 0.1303 (for observed data)

$\text{Goof} = S = \sqrt{\frac{\Sigma[w(F_o^2 - F_c^2)^2]}{n - p}}$ 1.030

Largest and mean Δ/σ	0.001, 0.000
Residual extrema in final difference map	-0.287 to 0.197 e Å^{-3}

10. Analytical data

2,2-difluorovinyl [1,1'-biphenyl]-2-carboxylate (2a):

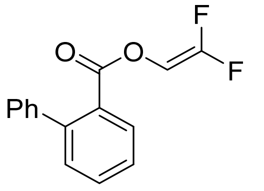

Colorless oil 41 mg (0.16 mmol, 79%). **^{1}H NMR** (400 MHz, Chloroform-*d*) δ 7.98 (d, J = 7.7 Hz, 1H), 7.63 (t, J = 7.6 Hz, 1H), 7.54 – 7.37 (m, 5H), 7.32 (d, J = 7.1 Hz, 2H), 6.83 (dd, J = 16.0, 3.1 Hz, 1H). **^{13}C NMR** (101 MHz, Chloroform-*d*) δ 164.6, 154.9 (dd, J = 291.3, 277.0 Hz), 143.8, 141.0, 132.5, 131.3, 130.7, 128.4, 128.3, 128.3,127.6, 127.5, 100.5 (dd, J = 60.3, 13.8 Hz). **^{19}F NMR** (376 MHz, Chloroform-*d*) δ -92.90 (dd, J = 62.8, 15.3 Hz, 1F), -114.02 (d, J = 62.6 Hz 1F). **HRMS (EI)** calculated for $C_{15}H_{10}F_2O_2$ [M]$^+$ 260.0649; found: 260.0644.

2,2-difluorovinyl 2-bromobenzoate (2b):

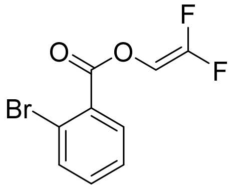

Colorless oil 33 mg (0.13 mmol, 63%). **^{1}H NMR** (400 MHz, Chloroform-*d*) δ 8.03 – 7.82 (m, 1H), 7.78 – 7.63 (m, 1H), 7.49 – 7.33 (m, 2H), 6.99 (dd, J = 16.0, 3.2 Hz, 1H). **^{13}C NMR** (101 MHz, Chloroform-*d*) δ 162.1, 155.3 (dd, J = 290.9, 278.2 Hz), 135.0, 133.8, 132.2, 129.6, 127.5, 122.8, 100.8 (dd, J = 60.4, 13.8 Hz). **^{19}F NMR** (376 MHz, Chloroform-*d*) δ -92.31 (dd, J = 62.8, 17.0 Hz, 1F), -113.62 (d, J = 63.5 Hz, 1F). **HRMS (EI)** calculated for $C_9H_5BrF_2O_2$ [M]$^+$ 261.9441; found: 261.9438.

2,2-difluorovinyl 2-bromo-5-methylbenzoate (2c):

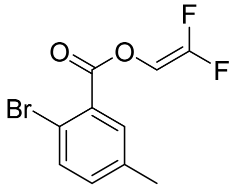

Colorless oil 39 mg (0.14 mmol, 70%). **^{1}H NMR** (400 MHz, Chloroform-*d*) δ 7.71 (d, *J* = 2.3 Hz, 1H), 7.57 (d, *J* = 8.1 Hz, 1H), 7.20 (dd, *J* = 8.3, 2.3 Hz, 1H), 6.97 (dd, *J* = 15.9, 3.0 Hz, 1H), 2.36 (s, 3H). **^{13}C NMR** (101 MHz, Chloroform-*d*) δ 162.3, 155.2 (dd, *J* = 290.9, 278.2 Hz), 137.7, 134.7, 132.7, 129.3, 119.4, 100.8 (dd, *J* = 60.5, 14.2 Hz), 20.9. **^{19}F NMR** (376 MHz, Chloroform-*d*) δ -92.38 (dd, *J* = 63.3, 16.7 Hz, 1F), -113.72 (d, *J* = 62.8 Hz, 1F). **HRMS (EI)** calculated for $C_{10}H_7BrF_2O_2$ $[M]^+$ 275.9597; found: 275.9592.

2,2-difluorovinyl 4-acetylbenzoate (2d):

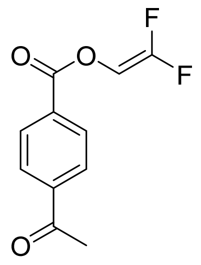

White solid 32 mg (0.14 mmol, 71%). **^{1}H NMR** (400 MHz, Chloroform-*d*) δ 8.17 (d, *J* = 8.3 Hz, 2H), 8.04 (d, *J* = 8.3 Hz, 2H), 7.01 (dd, *J* = 15.6, 3.2 Hz, 1H), 2.65 (s, 3H). **^{13}C NMR** (101 MHz, Chloroform-*d*) δ 197.4, 162.3, 155.3 (dd, *J* = 291.0, 277.7 Hz), 141.1, 131.9, 130.5, 128.5, 100.9 (dd, *J* = 60.5, 13.8 Hz), 27.0. **^{19}F NMR** (376 MHz, Chloroform-*d*) δ -92.46 (dd, *J* = 62.9, 15.2 Hz, 1F), -113.58 (dd, *J* = 62.9, 4.1 Hz, 1F). **HRMS (EI)** calculated for $C_{11}H_8F_2O_3$ $[M]^+$ 226.0442; found: 226.0437.

2,2-difluorovinyl 4-iodobenzoate (2e):

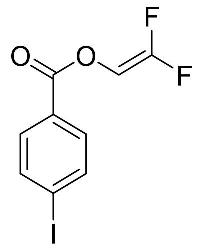

White solid 42 mg (0.14 mmol, 68%). **^{1}H NMR** (400 MHz, Chloroform-*d*) δ 7.85 (d, *J* = 8.6 Hz, 2H), 7.78 (d, *J* = 8.6 Hz, 2H), 6.97 (dd, *J* = 15.8, 3.1 Hz, 1H). **^{13}C NMR** (101 MHz, Chloroform-*d*) δ 162.8 (d, *J* = 1.7 Hz), 155.2 (dd, *J* = 290.7, 277.6 Hz), 138.3, 131.5, 127.7, 102.3, 100.8 (dd, *J* = 60.5, 13.8 Hz). **^{19}F NMR** (376 MHz, Chloroform-*d*) δ -92.93 (dd, *J* = 63.9, 15.7 Hz, 1F), -113.99 (dd, *J* = 63.9, 3.3 Hz, 1F). **HRMS (EI)** calculated for $C_9H_5F_2IO_2$ $[M]^+$ 309.9302; found: 309.9301.

2,2-difluorovinyl 4-fluoro-2-nitrobenzoate (2f):

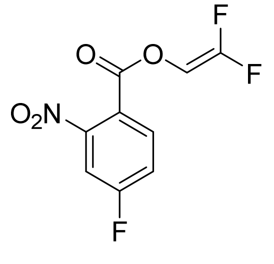

Colorless oil 32 mg (0.13 mmol, 65%). **^{1}H NMR** (400 MHz, Chloroform-*d*) δ 7.89 (dd, *J* = 8.6, 5.3 Hz, 1H), 7.64 (dd, *J* = 7.8, 2.6 Hz, 1H), 7.43 (m, 1H), 6.92 (dd, *J* = 15.2, 3.2 Hz, 1H). **^{13}C NMR** (101 MHz, Chloroform-*d*) δ 164.2 (d, *J* = 259.7 Hz), 160.7, 155.4 (dd, *J* = 291.8, 279.1 Hz), 150.1, 132.8 (d, *J* = 8.9 Hz), 121.2 (d, *J* = 4.0 Hz), 120.2 (d, *J* = 21.5 Hz), 112.6 (d, *J* = 27.0 Hz), 101.2 (dd, *J* = 60.8, 14.3 Hz), **^{19}F NMR** (376 MHz, Chloroform-*d*) δ -91.38 (dd, *J* = 59.5, 15.4 Hz, 1F), -101.35 (m, 1F), -112.44

(d, *J* = 60.5 Hz, 1F). **HRMS (EI)** calculated for $C_9H_4F_3NO_4$ $[M]^+$ 247.0092; found: 247.0088.

2,2-difluorovinyl 4-methoxybenzoate (2g):

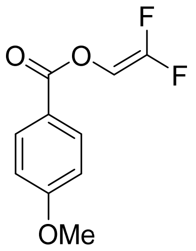

A mixture of colorless solid and liquid 38 mg (0.18 mmol, 88%). ^{1}**H NMR** (400 MHz, Chloroform-*d*) δ 8.04 (d, *J* = 8.9 Hz, 2H), 7.12 – 6.76 (m, 3H), 3.88 (s, 3H). ^{13}**C NMR** (101 MHz, Chloroform-*d*) δ 164.3, 162.9, 155.1(dd, *J* = 289.7, 276.8 Hz), 132.4, 120.5, 114.1, 100.7(dd, *J* = 60.5, 13.6 Hz), 55.7. ^{19}**F NMR** (376 MHz, Chloroform-*d*) δ -93.88 (dd, *J* = 65.0,15.2 Hz, 1F), -114.87(d, *J* = 69.4 Hz, 1F). **HRMS (EI)** calculated for $C_{10}H_8F_2O_3$ $[M]^+$ 214.0442; found: 214.0440.

2,2-difluorovinyl cinnamate (2h):

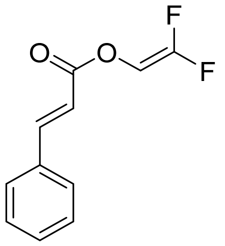

Colorless oil 26 mg (0.12 mmol, 62%). ^{1}**H NMR** (400 MHz, Chloroform-*d*) δ 7.81 (d, *J* = 16.0 Hz, 1H), 7.64 – 7.52 (m, 2H), 7.42 (m, 3H), 6.91 (dd, *J* = 16.0, 3.0 Hz, 1H), 6.49 (d, *J* = 16.0 Hz, 1H). ^{13}**C NMR** (101 MHz, Chloroform-*d*) δ 163.4, 155.0 (dd, *J* = 290.5, 277.0 Hz), 147.7, 134.0, 131.2, 129.2, 128.5, 115.7, 100.6 (dd, *J* = 59.9, 13.6 Hz), ^{19}**F NMR** (376 MHz, Chloroform-*d*) δ -93.53 (dd, *J* = 65.0, 15.2 Hz, 1F), -114.72 (d, *J* = 66.5 Hz, 1F). **HRMS (EI)** calculated for $C_{11}H_8F_2O_2$ $[M]^+$ 210.0492; found: 210.0488.

2,2-difluorovinyl 3,5-dichlorobenzoate (2i):

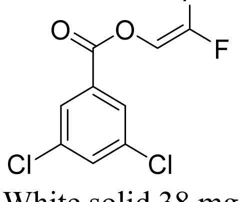

White solid 38 mg (0.15 mmol, 76%). ^{1}**H NMR** (400 MHz, Chloroform-*d*) δ 7.95 (d, *J* = 2.1 Hz, 2H), 7.61 (t, *J* = 2.0 Hz, 1H), 6.97 (dd, *J* = 15.5, 3.2 Hz, 1H). ^{13}**C NMR** (101 MHz, Chloroform-*d*) δ 161.0, 155.3 (dd, *J* = 291.9, 278.5 Hz), 136.0, 134.0, 131.0, 128.6, 100.9 (dd, *J* = 60.8, 13.7 Hz). ^{19}**F NMR** (376 MHz, Chloroform-*d*) δ -91.88 (dd, *J* = 60.8, 15.3 Hz, 1F), -113.07 (d, *J* = 58.7 Hz, 1F). **HRMS (EI)** calculated for $C_9H_4Cl_2F_2O_2$ $[M]^+$ 251.9556; found: 251.9553.

2,2-difluorovinyl 1-naphthoate (2j):

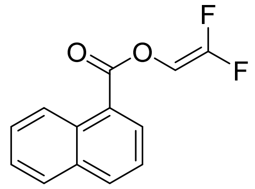

Colorless oil 30 mg (0.13 mmol, 64%). **^{1}H NMR** (400 MHz, Chloroform-*d*) δ 8.97 (d, *J* = 8.7 Hz, 1H), 8.34 (d, *J* = 7.3 Hz, 1H), 8.10 (d, *J* = 8.2 Hz, 1H), 7.91 (d, *J* = 8.1 Hz, 1H), 7.67 (t, *J* = 7.7 Hz, 1H), 7.56 (dt, *J* = 15.5, 7.7 Hz, 2H), 7.09 (dd, *J* = 16.1, 3.0 Hz, 1H). **^{13}C NMR** (101 MHz, Chloroform-*d*) 163.5, 155.2 (dd, *J* = 290.1, 277.6 Hz), 134.9, 134.0, 131.7, 131.6, 128.9, 128.6, 126.7, 125.6, 124.6, 124.5, 100.8 (dd, *J* = 60.4, 13.6 Hz), **^{19}F NMR** (376 MHz, Chloroform-*d*) δ -93.22 (dd, *J* = 65.0, 15.3 Hz, 1F), -114.30 (d, *J* = 65.1 Hz, 1F). **HRMS (EI)** calculated for $C_{13}H_8F_2O_2$ $[M]^+$ 234.0492; found: 234.0487.

2,2-difluorovinyl 2-(2-fluoro-[1,1'-biphenyl]-4-yl)propanoate (2k):

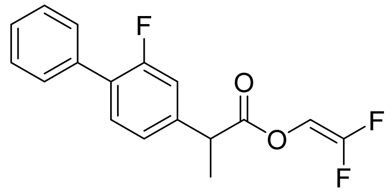

Colorless oil 42 mg (0.14 mmol, 69%). **^{1}H NMR** (400 MHz, Chloroform-*d*) δ 7.64 – 7.52 (m, 2H), 7.49 – 7.33 (m, 4H), 7.22 – 7.07 (m, 2H), 6.76 (dd, *J* = 15.8, 3.1 Hz, 1H), 3.90 (q, *J* = 7.2 Hz, 1H), 1.61 (d, *J* = 7.2 Hz, 3H). **^{13}C NMR** (101 MHz, Chloroform-*d*) δ 170.6 159.9 (d, *J* = 248.6 Hz), 155.0 (dd, *J* = 290.2, 277.5 Hz), 140.5 (d, *J* = 7.6 Hz), 135.5, 131.2 (d, *J* = 4.2 Hz), 129.1 (d, *J* = 2.6 Hz), 128.6, 128.4, 127.9, 123.7 (d, *J* = 3.7 Hz), 115.4 (d, *J* = 24.0 Hz), 100.7 (dd, *J* = 59.8, 13.8 Hz), 44.7, 18.4. **^{19}F NMR** (376 MHz, Chloroform-*d*) δ -92.98 (dd, *J* = 62.9, 15.2 Hz, 1F), -114.30 (d, *J* = 61.4 Hz, 1F), -117.0 (t, *J* = 10.0 Hz, 1F). **HRMS (EI)** calculated for $C_{17}H_{13}F_3O_2$ $[M]^+$ 306.0868; found: 306.0862.

bis(2,2-difluorovinyl) terephthalate (2l):

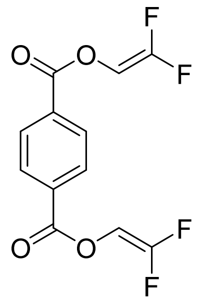

White solid 15 mg (0.05 mmol, 25%). **^{1}H NMR** (400 MHz, Chloroform-*d*) δ 8.20 (s, 4H), 7.02 (dd, *J* = 15.6, 2.9 Hz, 2H). **^{13}C NMR** (101 MHz, Chloroform-*d*) δ 162.1, 155.3 (d, *J* = 294.3, 279.0 Hz), 132.9, 130.4, 100.9 (dd, *J* = 60.5, 13.9 Hz). **^{19}F NMR**

(376 MHz, Chloroform-*d*) δ -92.30 (dd, *J* = 62.7, 15.7 Hz, 2F), -113.43 (d, *J* = 62.5 Hz, 2F). **HRMS (EI)** calculated for $C_{12}H_6F_4O_4$ $[M]^+$ 290.0202; found: 290.0200.

N-(2,2-difluorovinyl)-1-naphthamide (3a):

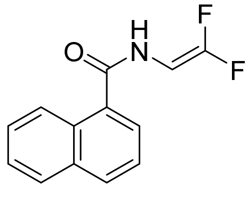

White solid 38 mg (0.16 mmol, 82%). **^{1}H NMR** (400 MHz, Chloroform-*d*) δ 8.25 (d, *J* = 7.2 Hz, 1H), 7.93 (d, *J* = 8.3 Hz, 1H), 7.89 – 7.80 (m, 1H), 7.66 – 7.47 (m, 3H), 7.42 (dd, *J* = 8.4, 7.0 Hz, 1H), 7.19 (s, 1H), 6.46 (dd, *J* = 20.7, 9.6 Hz, 1H). **^{13}C NMR** (101 MHz, Chloroform-*d*) δ 166.7 (d, *J* = 2.9 Hz), 153.4 (dd, *J* = 285.3, 280.1 Hz), 133.8, 132.5, 131.7, 130.2, 128.5, 127.6, 126.8, 125.5, 125.2, 124.7, 83.5 (dd, *J* = 48.6, 14.8 Hz). **^{19}F NMR** (376 MHz, Chloroform-*d*) δ -93.49 (dd, *J* = 65.5, 20.7 Hz, 1F), -108.28 (d, *J* = 65.7 Hz, 1F). **HRMS (EI)** calculated for $C_{13}H_9F_2NO$ $[M]^+$ 233.0652; found: 233.0647.

N-(2,2-difluorovinyl)-2-naphthamide (3b):

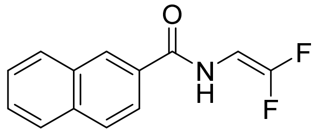

White solid 37 mg (0.16 mmol, 79%). **^{1}H NMR** (400 MHz, Chloroform-*d*) δ 8.30 (s, 1H), 8.06 – 7.73 (m, 4H), 7.57 (dt, *J* = 15.5, 6.9 Hz, 2H), 7.40 (d, *J* = 9.3 Hz, 1H), 6.48 (dd, *J* = 20.8, 9.5 Hz, 1H). **^{13}C NMR** (101 MHz, Chloroform-*d*) δ 164.9 (d, *J* = 2.0 Hz), 153.5 (dd, *J* = 285.1, 280.2 Hz), 135.1, 132.6, 130.2, 129.1, 128.9, 128.3, 128.1, 127.9, 127.2, 123.4, 83.7 (dd, *J* = 48.9, 14.6 Hz). **^{19}F NMR** (376 MHz, Chloroform-*d*) δ -93.77 (dd, *J* = 66.9, 21.3 Hz, 1F), -108.34 (d, *J* = 66.6 Hz, 1F). **HRMS (EI)** calculated for $C_{13}H_9F_2NO$ $[M]^+$ 233.0652; found: 233.0646.

4-(tert-butyl)-N-(2,2-difluorovinyl)benzamide (3c):

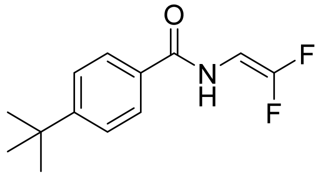

White solid 24 mg (0.10 mmol, 51%). **^{1}H NMR** (400 MHz, Chloroform-*d*) δ 7.74 (d, *J* = 8.7 Hz, 2H), 7.47 (d, *J* = 8.3 Hz, 2H), 7.16 (d, *J* = 9.3 Hz,1H), 6.42 (dd, *J* = 21.0, 9.9 Hz, 1H), 1.34 (s, 9H). **^{13}C NMR** (101 MHz, Chloroform-*d*) δ 164.7, 156.1, 153.4 (dd, *J* = 284.0, 279.5 Hz), 130.2, 127.1, 125.9, 83.7 (dd, *J* = 48.5, 14.5 Hz), 35.2, 31.2. **^{19}F NMR** (376 MHz, Chloroform-*d*) δ -94.35 (dd, *J* = 67.2, 21.7 Hz, 1F), -108.81 (d, *J* = 67.3 Hz, 1F). **HRMS (EI)** calculated for $C_{13}H_{15}F_2NO$ $[M]^+$ 239.1122; found: 239.1120.

N-(2,2-difluorovinyl)-4-methylbenzamide (3d):

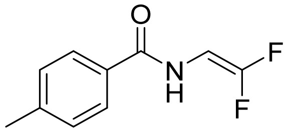

White solid 22 mg (0.11 mmol, 56%). **^{1}H NMR** (400 MHz, Chloroform-*d*) δ 7.69 (d, *J* = 8.2 Hz, 2H), 7.25 (d, *J* = 7.9 Hz, 2H), 7.19 (d, *J* = 9.3 Hz 1H), 6.40 (ddd, *J* = 20.7, 9.5, 1.3 Hz, 1H), 2.41 (s, 3H). **^{13}C NMR** (101 MHz, Chloroform-*d*) δ 164.7, 153.4 (dd, *J* = 284.5, 279.3 Hz), 143.1, 130.2, 129.6, 127.2, 83.6 (dd, *J* = 48.8, 14.8 Hz), 21.6. **^{19}F NMR** (376 MHz, Chloroform-*d*) δ -94.31 (dd, *J* = 67.6, 19.9 Hz, 1F), -108.80 (d, *J* = 68.4 Hz, 1F). **HRMS (EI)** calculated for $C_{10}H_9F_2NO$ $[M]^+$ 197.0652; found: 197.0649.

2-chloro-N-(2,2-difluorovinyl)benzamide (3e):

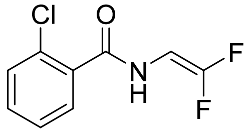

White solid 19 mg (0.09 mmol, 44%). **^{1}H NMR** (400 MHz, Chloroform-*d*) δ 7.77 (d, *J* = 7.7 Hz, 1H), 7.55 – 7.31 (m, 4H), 6.41 (dd, *J* = 20.5, 9.5 Hz, 1H). **^{13}C NMR** (101 MHz, Chloroform-*d*) δ 163.4, 153.6 (dd, *J* = 285.9, 280.4 Hz), 133.0, 132.4, 131.1, 130.9, 130.7, 127.5, 83.3 (dd, *J* = 48.9, 14.9 Hz). **^{19}F NMR** (376 MHz, Chloroform-*d*) δ -93.38 (dd, *J* = 64.3, 20.5 Hz, 1F), -107.78 (d, *J* = 65.1 Hz, 1F). **HRMS (EI)** calculated for $C_9H_6ClF_2NO$ $[M]^+$ 217.0106; found: 217.0102.

3-chloro-N-(2,2-difluorovinyl)benzamide (3f):

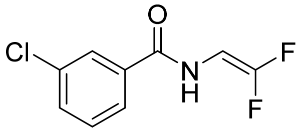

White solid 42 mg (0.19 mmol, 96%). **^{1}H NMR** (400 MHz, Chloroform-*d*) δ 7.78 (s, 1H), 7.66 (d, *J* = 7.7 Hz, 1H), 7.52 (dd, *J* = 8.2, 1.8 Hz, 1H), 7.40 (t, *J* = 7.9 Hz, 1H), 7.21 (d, *J* = 9.3 Hz, 1H), 6.38 (dd, *J* = 20.6, 9.4 Hz, 1H). **^{13}C NMR** (101 MHz, Chloroform-*d*) δ 163.5, 153.5 (dd, *J* = 285.2, 280.4 Hz), 135.2, 134.8, 132.5, 130.3, 127.6, 125.3, 83.5 (dd, *J* = 48.9, 14.8 Hz). **^{19}F NMR** (376 MHz, Chloroform-*d*) δ -93.18 (dd, *J* = 65.1, 19.5 Hz, 1F), -107.62 (d, *J* = 64.8 Hz, 1F). **HRMS (EI)** calculated for $C_9H_6ClF_2NO$ $[M]^+$ 217.0106; found:217.0102.

4-chloro-N-(2,2-difluorovinyl)benzamide (3g):

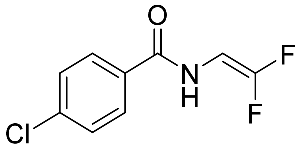

White solid 23 mg (0.10 mmol, 52%). **^{1}H NMR** (400 MHz, Chloroform-*d*) δ 7.73 (d, *J* = 8.4 Hz, 2H), 7.43 (d, *J* = 8.4 Hz, 2H), 7.19 (d, *J* = 4.2 Hz, 1H), 6.38 (ddd, *J* = 20.5,

9.4, 1.6 Hz, 1H). **^{13}C NMR** (101 MHz, Chloroform-*d*) δ 163.7 (d, *J* = 2.0 Hz), 153.5 (dd, *J* = 285.1, 280.7 Hz), 138.8, 131.4, 129.3, 128.7, 83.5 (dd, *J* = 49.1, 14.5 Hz). **^{19}F NMR** (376 MHz, Chloroform-*d*) δ -93.48 (dd, *J* = 65.2, 21.0 Hz, 1F), -107.89 (d, *J* = 67.0 Hz, 1F). **HRMS (EI)** calculated for $C_9H_6ClF_2NO$ $[M]^+$ 217.0106; found: 217.0102.

N-(2,2-difluorovinyl)-4-fluorobenzamide (3h):

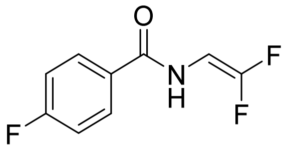

White solid 31 mg (0.16 mmol, 78%). **^{1}H NMR** (400 MHz, Chloroform-*d*) δ 7.91 – 7.63 (m, 2H), 7.22 (d, *J* = 9.4 Hz, 1H), 7.16 – 7.05 (m, 2H), 6.37 (ddd, *J* = 20.3, 9.3, 1.6 Hz, 1H). **^{13}C NMR** (101 MHz, Chloroform-*d*) δ 165.3 (d, *J* = 253.4 Hz), 163.8 (d, *J* = 2.0 Hz), 153.5 (dd, *J* = 284.4, 280.4 Hz), 129.7 (d, *J* = 8.8 Hz), 129.2 (d, *J* = 2.8 Hz), 116.1 (d, *J* = 21.7 Hz), 83.6 (dd, *J* = 49.0, 14.5 Hz). **^{19}F NMR** (376 MHz, Chloroform-*d*) δ -93.7 (dd, *J* = 65.2, 19.6 Hz, 1F), -106.30 – -106.37 (m, 1F), -108.11 (d, *J* = 67.1 Hz, 1F). **HRMS (EI)** calculated for $C_9H_6F_3NO$ $[M]^+$ 201.0401; found: 201.0397.

N-(2,2-difluorovinyl)-4-(trifluoromethoxy)benzamide (3i):

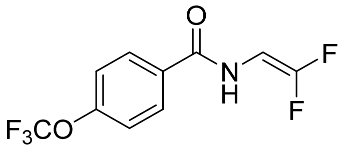

White solid 52 mg (0.19 mmol, 98%). **^{1}H NMR** (400 MHz, Chloroform-*d*) δ 7.85 (d, *J* = 8.5 Hz, 2H), 7.21-7.37 (m, 3H), 6.38 (dd, *J* = 20.5, 9.4 Hz, 1H). **^{13}C NMR** (101 MHz, Chloroform-*d*) δ 163.6 (d, *J* = 2.0 Hz), 153.5 (dd, *J* = 285.1, 280.5 Hz), 152.2, 131.4, 129.2, 120.9, 120.4 (q, *J* = 258.9 Hz), 83.5 (dd, *J* = 49.1, 14.7 Hz). **^{19}F NMR** (376 MHz, Chloroform-*d*) δ -57.68 (s, 3F), -93.36 (dd, *J* = 65.0, 21.7 Hz, 1F), -107.75 (d, *J* = 65.0 Hz, 1F). **HRMS (EI)** calculated for $C_{10}H_6F_5NO_2$ $[M]^+$ 267.0319; found: 267.0315.

N-(2,2-difluorovinyl)-2-(naphthalen-1-yl)acetamide (3j):

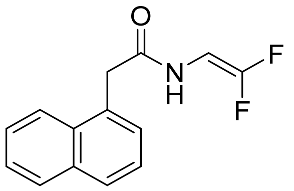

White solid 17 mg (0.07 mmol, 35%). **^{1}H NMR** (400 MHz, Chloroform-*d*) δ 7.96 – 7.89 (m, 2H), 7.86 (d, *J* = 8.1 Hz, 1H), 7.60 – 7.50 (m, 2H), 7.50 – 7.39 (m, 2H), 6.39 (d, *J* = 9.2 Hz, 1H), 6.12 (ddd, *J* = 20.5, 9.3, 1.5 Hz, 1H), 4.09 (s, 2H). **^{13}C NMR** (101 MHz, Chloroform-*d*) δ 168.5 (d, *J* = 2.0 Hz), 153.0 (dd, *J* = 285.1, 279.4 Hz), 134.2, 132.0, 130.1, 129.1, 128.5, 127.2, 126.5, 125.8, 123.6, 83.0 (dd, *J* = 48.9, 15.0 Hz), 41.5. **^{19}F NMR** (376 MHz, Chloroform-*d*) δ -94.12 (dd, *J* = 65.5, 20.0 Hz, 1F), -108.70

(d, J = 67.1 Hz, 1F). **HRMS (EI)** calculated for $C_{14}H_{11}F_2NO$ $[M]^+$ 247.0809; found: 247.0805.

5-chloro-N-(2,2-difluorovinyl)thiophene-2-carboxamide (3k):

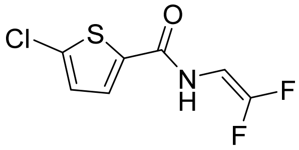

White solid 26 mg (0.12 mmol, 58%). **^{1}H NMR** (400 MHz, Chloroform-*d*) δ 7.37 (s, 1H), 7.01 (s, 1H), 6.93 (s, 1H), 6.32 (dd, J = 20.5, 9.4 Hz, 1H). **^{13}C NMR** (101 MHz, Chloroform-*d*) δ 158.2 (d, J = 3.0 Hz), 153.3 (dd, J = 285.4, 280.1 Hz), 137.0, 135.8, 128.3, 127.4, 83.2 (dd, J = 49.4, 14.8 Hz). **^{19}F NMR** (376 MHz, Chloroform-*d*) δ -93.46 (dd, J = 65.3, 20.5 Hz, 1F), -107.84 (d, J = 65.4 Hz, 1F). **HRMS (EI)** calculated for $C_7H_4ClF_2NOS$ $[M]^+$ 222.9670; found: 222.9666.

5-fluoro-2-(naphthalen-1-yl)oxazole (4a):

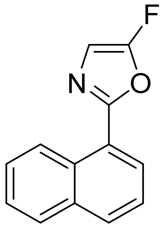

Light yellow oil 29 mg (0.14 mmol, 69%). **^{1}H NMR** (400 MHz, Chloroform-*d*) δ 9.22 (d, J = 8.4 Hz, 1H), 8.08 (d, J = 7.2 Hz, 1H), 7.93 (dd, J = 19.0, 8.1 Hz, 2H), 7.65 (t, J = 7.7 Hz, 1H), 7.61-7.49 (m, 2H), 6.62 (d, J = 8.9 Hz, 1H). **^{13}C NMR** (101 MHz, Chloroform-*d*) δ 158.6 (d, J = 285.1 Hz), 152.5 (d, J = 5.8 Hz), 134.0, 131.4, 130.1, 128.7, 127.8, 127.5, 126.5, 126.0, 125.0, 123.2, 100.7 (d, J = 12.5 Hz). **^{19}F NMR** (376 MHz, Chloroform-*d*) δ -131.23 (d, J = 8.6 Hz). **HRMS (EI)** calculated for $C_{13}H_8FNO$ $[M]^+$ 213.0590; found: 213.0586.

5-fluoro-2-(naphthalen-2-yl)oxazole (4b):

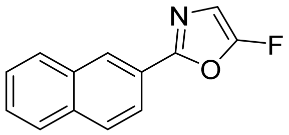

White solid 22 mg (0.10 mmol, 51%). **^{1}H NMR** (400 MHz, Chloroform-*d*) δ 8.40 (s, 1H), 8.02 (d, J = 9.7 Hz, 1H), 7.94 – 7.88 (m, 2H), 7.87 – 7.82 (m, 1H), 7.61 – 7.47 (m, 2H), 6.53 (d, J = 8.8 Hz, 1H). **^{13}C NMR** (101 MHz, Chloroform-*d*) δ 158.6 (d, J = 286.1 Hz), 152.8 (d, J = 6.1 Hz), 134.2, 133.0, 128.9, 128.8, 128.0, 127.5, 127.0, 125.7 (d, J = 1.6 Hz), 124.2, 122.6, 101.0 (d, J = 12.3 Hz). **^{19}F NMR** (376 MHz, Chloroform-*d*) δ -130.11 (d, J = 8.7 Hz). **HRMS (EI)** calculated for $C_{13}H_8FNO$ $[M]^+$ 213.0590; found: 213.0586.

5-fluoro-2-phenyloxazole (4c):

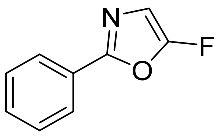

Colorless oil 27 mg (0.18 mmol, 83%). **^{1}H NMR** (400 MHz, Chloroform-*d*) δ 7.98 – 7.89 (m, 2H), 7.53 – 7.40 (m, 3H), 6.48 (d, *J* = 8.8 Hz, 1H). **^{13}C NMR** (101 MHz, Chloroform-*d*) δ 158.5 (d, *J* = 285.6 Hz), 152.6 (d, *J* = 7.5 Hz), 130.6, 129.0, 126.9, 125.8 (d, *J* = 1.4 Hz), 100.8 (d, *J* = 12.4 Hz). **^{19}F NMR** (376 MHz, Chloroform-*d*) δ -130.38 (d, *J* = 8.9 Hz). **HRMS (EI)** calculated for C_9H_6FNO $[M]^+$ 163.0433; found: 163.0430.

2-(4-(tert-butyl)phenyl)-5-fluorooxazole (4d):

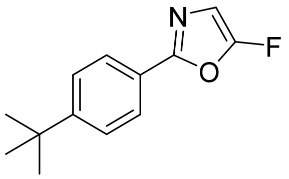

Light yellow oil 39 mg (0.18 mmol, 90%). **^{1}H NMR** (400 MHz, Chloroform-*d*) δ 7.86 (d, *J* = 7.4 Hz, 2H), 7.47 (d, *J* = 7.2 Hz, 2H), 6.45 (d, *J* = 8.8 Hz, 1H), 1.34 (s, 9H). **^{13}C NMR** (101 MHz, Chloroform-*d*) δ 158.4 (d, *J* = 285.1 Hz), 154.0, 152.8 (d, *J* = 6.5 Hz), 125.9, 125.6, 124.2, 100.5 (d, *J* = 12.5 Hz), 35.1, 31.3. **^{19}F NMR** (376 MHz, Chloroform-*d*) δ -130.76 (d, *J* = 8.6 Hz). **HRMS (EI)** calculated for $C_{13}H_{14}FNO$ $[M]^+$ 219.1059; found: 219.1056.

5-fluoro-2-(4-methoxyphenyl)oxazole (4e):

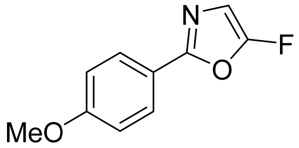

Light yellow oil 25 mg (0.13 mmol, 64%). **^{1}H NMR** (400 MHz, Chloroform-*d*) δ 7.85 (d, *J* = 8.1 Hz, 2H), 6.95 (d, *J* = 8.2 Hz, 2H), 6.41 (d, *J* = 8.7 Hz, 1H), 3.85 (s, 3H). **^{13}C NMR** (101 MHz, Chloroform-*d*) δ 161.5, 158.4 (d, *J* = 284.8 Hz), 152.8 (d, *J* = 6.3 Hz), 127.5 (d, *J* = 1.0 Hz), 119.8, 114.4, 100.3 (d, *J* = 12.4 Hz), 55.5. **^{19}F NMR** (376 MHz, Chloroform-d) δ -131.56 (d, *J* = 8.7 Hz). **HRMS (EI)** calculated for $C_{10}H_8FNO_2$ $[M]^+$ 193.0539; found: 193.0537.

5-fluoro-2-(p-tolyl)oxazole (4f):

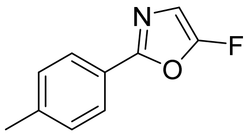

Colorless oil 23 mg (0.13 mmol, 65%). **^{1}H NMR** (400 MHz, Chloroform-*d*) δ 7.82 (d, *J* = 8.2 Hz, 2H), 7.25 (d, *J* = 7.9 Hz, 2H), 6.44 (d, *J* = 8.7 Hz, 1H), 2.40 (s, 3H). **^{13}C**

NMR (101 MHz, Chloroform-*d*) δ 158.4 (d, *J* = 285.1 Hz), 152.8 (d, *J* = 5.7 Hz), 140.9, 129.7, 125.7 (d, *J* = 1.1 Hz), 124.3, 100.5 (d, *J* = 12.4 Hz), 21.7. **^{19}F NMR** (376 MHz, Chloroform-*d*) δ -130.86 (d, *J* = 8.6 Hz). **HRMS (EI)** calculated for $C_{10}H_8FNO$ $[M]^+$ 177.0590; found: 177.0588.

5-fluoro-2-(o-tolyl)oxazole (4g):

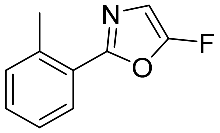

Colorless oil 32 mg (0.18 mmol, 90%). **^{1}H NMR** (400 MHz, Chloroform-*d*) δ 7.86 (d, *J* = 8.3 Hz, 1H), 7.46 – 7.19 (m, 3H), 6.50 (dd, *J* = 8.8, 2.6 Hz, 1H), 2.65 (s, 3H). **^{13}C NMR** (101 MHz, Chloroform-*d*) δ 158.5 (d, *J* = 284.6 Hz), 152.9 (d, *J* = 7.5 Hz), 137.4, 131.8, 130.1, 128.4, 126.2, 125.9, 100.4 (d, *J* = 12.4 Hz), 21.9. **^{19}F NMR** (376 MHz, Chloroform-*d*) δ -131.42 (d, *J* = 8.5 Hz). **HRMS (EI)** calculated for $C_{10}H_8FNO$ $[M]^+$ 177.0590; found: 177.0588.

5-fluoro-2-(m-tolyl)oxazole (4h):

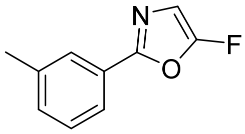

Colorless oil 27 mg (0.15 mmol, 75%). **^{1}H NMR** (400 MHz, Chloroform-*d*) δ 7.76 (s, 1H), 7.72 (d, *J* = 7.8 Hz, 1H), 7.34 (t, *J* = 7.6 Hz, 1H), 7.29 – 7.19 (m, 1H), 6.46 (d, *J* = 8.6 Hz, 1H), 2.41(s, 3H). **^{13}C NMR** (101 MHz, Chloroform-*d*) δ 158.5 (d, *J* = 285.9 Hz), 152.8 (d, *J* = 5.9 Hz), 138.8, 131.4, 128.9, 126.9, 126.4 (d, *J* = 1.0 Hz), 123.0, 100.7 (d, *J* = 12.4 Hz), 21.5. **^{19}F NMR** (376 MHz, Chloroform-*d*) δ -130.51 (d, *J* = 8.7 Hz). **HRMS (EI)** calculated for $C_{10}H_8FNO$ $[M]^+$ 177.0590; found: 177.0588.

5-fluoro-2-(4-(trifluoromethyl)phenyl)oxazole (4i):

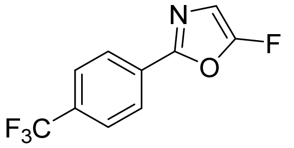

.

Colorless oil 37 mg (0.16 mmol, 81%). **^{1}H NMR** (400 MHz, Chloroform-*d*) δ 8.04 (d, *J* = 8.2 Hz, 2H), 7.71 (d, *J* = 8.2 Hz, 2H), 6.54 (d, *J* = 8.7 Hz, 1H). **^{13}C NMR** (101 MHz, Chloroform-*d*) δ 158.8 (d, *J* = 287.8 Hz), 151.2 (d, *J* = 7.3 Hz), 132.2 (q, *J* = 32.8 Hz), 130.0, 126.3 – 125.8 (m, 2C), 123.9 (q, *J* = 272.3 Hz), 101.5 (d, *J* = 12.4 Hz). **^{19}F NMR** (376 MHz, Chloroform-*d*) δ -62.81 (s, 3F), -128.62 (d, *J* = 8.4 Hz, 1F). **HRMS (EI)** calculated for $C_{10}H_5F_4NO$ $[M]^+$ 231.0307; found: 231.0302.

methyl 4-(5-fluorooxazol-2-yl)benzoate (4j):

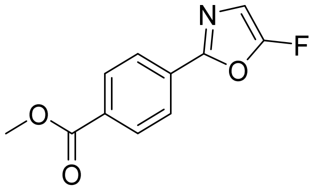

Light yellow solid 20 mg (0.09 mmol, 45%). **^{1}H NMR** (400 MHz, Chloroform-*d*) δ 8.10 (d, *J* = 8.2 Hz, 2H), 7.98 (d, *J* = 8.0 Hz, 2H), 6.52 (d, *J* = 8.8 Hz, 1H), 3.93(s, 3H). **^{13}C NMR** (101 MHz, Chloroform-*d*) δ 166.4, 158.8 (d, *J* = 287.4 Hz), 151.6 (d, *J* = 6.6 Hz), 131.7, 130.6, 130.2, 125.6, 101.5 (d, *J* = 12.4 Hz), 52.5. **^{19}F NMR** (376 MHz, Chloroform-*d*) δ -128.86 (d, *J* = 8.8 Hz). **HRMS (EI)** calculated for $C_{11}H_8FNO_3$ $[M]^+$ 221.0488; found: 221.0487.

5-fluoro-2-(4-fluorophenyl)oxazole (4k):

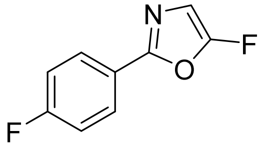

Colorless oil 26 mg (0.14 mmol, 72%). **^{1}H NMR** (400 MHz, Chloroform-*d*) δ 7.92 (dd, *J* = 8.9, 5.3 Hz, 2H), 7.14 (t, *J* = 8.5 Hz, 2H), 6.46 (d, *J* = 8.8 Hz, 1H). **^{13}C NMR** (101 MHz, Chloroform-*d*) δ 164.2 (d, *J* = 251.2 Hz), 158.5 (d, *J* = 285.7 Hz), 151.8 (d, *J* = 6.5 Hz), 127.9 (d, *J* = 8.5 Hz), 123.3, 116.2 (d, *J* = 22.4 Hz), 100.8 (d, *J* = 12.5 Hz). **^{19}F NMR** (376 MHz, Chloroform-*d*) δ -109.00 – -109.16 (m, 1F), -130.34 (d, *J* = 8.7 Hz, 1F). **HRMS (EI)** calculated for $C_9H_5F_2NO$ $[M]^+$ 181.0339; found: 181.0335.

5-fluoro-2-(4-(trifluoromethoxy)phenyl)oxazole (4l):

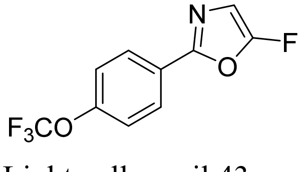

Light yellow oil 43 mg (0.18 mmol, 88%). **^{1}H NMR** (400 MHz, Chloroform-*d*) δ 7.96 (d, *J* = 9.0 Hz, 2H), 7.29 (d, *J* = 8.4 Hz, 2H), 6.49 (d, *J* = 8.7 Hz, 1H). **^{13}C NMR** (101 MHz, Chloroform-*d*) δ 158.7 (d, *J* = 286.9 Hz), 151.4 (d, *J* = 6.9 Hz), 150.8, 127.5, 125.6, 121.3, 120.5 (q, *J* = 258.1 Hz), 101.1 (d, *J* = 12.3 Hz). **^{19}F NMR** (376 MHz, Chloroform-*d*) δ -57.82 (s, 3F), -129.89 (d, *J* = 8.5 Hz, 1F). **HRMS (EI)** calculated for $C_{10}H_5F_4NO_2$ $[M]^+$ 247.0256; found: 247.0251.

2-(4-chlorophenyl)-5-fluorooxazole (4m):

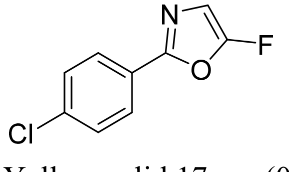

Yellow solid 17 mg (0.09 mmol, 44%). **^{1}H NMR** (400 MHz, Chloroform-*d*) δ 7.85 (d, *J* = 8.7 Hz, 2H), 7.42 (d, *J* = 8.7 Hz, 2H), 6.47 (d, *J* = 8.8 Hz, 1H). **^{13}C NMR** (101 MHz,

Chloroform-*d*) δ 158.5 (d, *J* = 286.7 Hz), 151.7 (d, *J* = 6.8 Hz), 136.7, 129.3, 127.0 (d, *J* = 1.2 Hz), 125.4, 101.0 (d, *J* = 12.3 Hz). **^{19}F NMR** (376 MHz, Chloroform-*d*) δ -129.89 (d, *J* = 8.8 Hz). **HRMS (EI)** calculated for C_9H_5FClNO $[M]^+$ 197.0044; found: 197.0041.

2-(2-chlorophenyl)-5-fluorooxazole (4n):

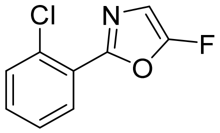

Colorless oil 19 mg (0.10 mmol, 48%). **^{1}H NMR** (400 MHz, Chloroform-*d*) δ 7.96 – 7.82 (m, 1H), 7.57 – 7.44 (m, 1H), 7.42 – 7.31 (m, 2H), 6.57 (d, *J* = 8.8 Hz, 1H). **^{13}C NMR** (101 MHz, Chloroform-*d*) δ 158.6 (d, *J* = 286.6 Hz), 150.3 (d, *J* = 7.1 Hz), 132.4, 131.4, 131.3, 130.5, 127.1, 125.6, 101.0 (d, *J* = 12.1 Hz). **^{19}F NMR** (376 MHz, Chloroform-*d*) δ -129.61 (d, *J* = 8.8 Hz). **HRMS (EI)** calculated for C_9H_5FClNO $[M]^+$ 197.0044; found: 197.0041.

2-(3-chlorophenyl)-5-fluorooxazole (4o):

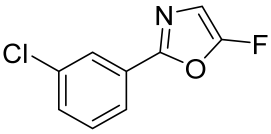

Colorless oil 33 mg (0.17 mmol, 85%). **^{1}H NMR** (400 MHz, Chloroform-*d*) δ 7.92 (s, 1H), 7.81 (d, *J* = 7.3 Hz, 1H), 7.53 – 7.32 (m, 2H), 6.49 (d, *J* = 8.7 Hz, 1H). **^{13}C NMR** (101 MHz, Chloroform-*d*) δ 158.6 (d, *J* = 287.4 Hz), 151.2 (d, *J* = 6.8 Hz), 135.1, 130.6, 130.3, 128.5, 125.9, 123.8, 101.2 (d, *J* = 12.3 Hz). **^{19}F NMR** (376 MHz, Chloroform-*d*) δ -129.45 (d, *J* = 8.7 Hz). **HRMS (EI)** calculated for C_9H_5FClNO $[M]^+$ 197.0044; found: 197.0041.

2-(3-bromophenyl)-5-fluorooxazole (4p):

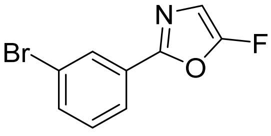

Colorless oil 43 mg (0.18 mmol, 90%). **^{1}H NMR** (400 MHz, Chloroform-*d*) δ 8.08 (s, 1H), 7.85 (d, *J* = 8.7 Hz, 1H), 7.57 (d, *J* = 8.2 Hz, 1H), 7.32 (t, *J* = 7.9 Hz, 1H), 6.49 (d, *J* = 8.7 Hz, 1H). **^{13}C NMR** (101 MHz, Chloroform-*d*) δ 158.6 (d, *J* = 287.1 Hz), 151.1 (d, *J* = 6.8 Hz), 133.5, 130.5, 128.8, 124.3, 123.1, 101.2 (d, *J* = 12.4 Hz). **^{19}F NMR** (376 MHz, Chloroform-*d*) δ -129.28 (d, *J* = 8.7 Hz). **HRMS (EI)** calculated for C_9H_5FBrNO $[M]^+$ 240.9539; found: 240.9537.

5-fluoro-2-(3-iodophenyl)oxazole (4q):

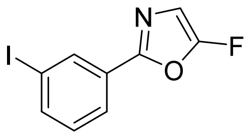

White solid 29 mg (0.10 mmol, 50%). **^{1}H NMR** (400 MHz, Chloroform-*d*) δ 8.28 (s, 1H), 7.88 (d, *J* = 7.9 Hz, 1H), 7.77 (d, *J* = 7.9 Hz, 1H), 7.18 (t, *J* = 7.9 Hz, 1H), 6.49 (d, *J* = 8.8 Hz, 1H). **^{13}C NMR** (101 MHz, Chloroform-*d*) δ 158.6 (d, *J* = 287.4 Hz), 150.9 (d, *J* = 9.2 Hz), 139.4, 134.5, 130.6, 128.7, 124.8, 101.2 (d, *J* = 12.3 Hz), 94.5. **^{19}F NMR** (376 MHz, Chloroform-*d*) δ -129.38 (d, *J* = 8.8 Hz). **HRMS (EI)** calculated for C_9H_5FINO $[M]^+$ 288.9400; found: 288.9397.

5-fluoro-2-(3-nitrophenyl)oxazole (4r):

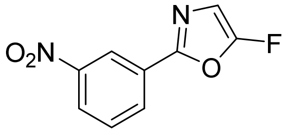

White solid 37 mg (0.18 mmol, 89%). **^{1}H NMR** (400 MHz, Chloroform-*d*) δ 8.76 (s, 1H), 8.27 (dd, *J* = 16.2, 8.0 Hz, 2H), 7.65 (t, *J* = 8.0 Hz, 1H), 6.56 (d, *J* = 8.6 Hz, 1H). **^{13}C NMR** (101 MHz, Chloroform-*d*) δ 158.8 (d, *J* = 288.7 Hz), 150.3 (d, *J* = 6.5 Hz), 148.8, 131.2, 130.2, 128.5, 124.9, 120.7, 101.7 (d, *J* = 12.3 Hz). **^{19}F NMR** (376 MHz, Chloroform-*d*) δ -128.15 (d, *J* = 8.2 Hz). **HRMS (EI)** calculated for $C_9H_5FN_2O_3$ $[M]^+$ 208.0284; found: 208.0281.

2-([1,1'-biphenyl]-2-yl)-5-fluorooxazole (4s):

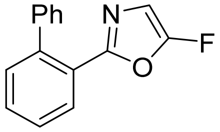

A mixture of colorless solid and oil 43 mg (0.18 mmol, 90%). **^{1}H NMR** (400 MHz, Chloroform-*d*) δ 7.86 (d, *J* = 7.6 Hz, 1H), 7.51 (t, *J* = 7.5 Hz, 1H), 7.45 (d, *J* = 7.6 Hz, 1H), 7.43 – 7.31 (m, 4H), 7.30 – 7.19 (m, 2H), 6.35 (d, *J* = 8.7 Hz, 1H). **^{13}C NMR** (101 MHz, Chloroform-*d*) δ 158.4 (d, *J* = 285.8 Hz), 152.7 (d, *J* = 6.2 Hz), 141.7, 140.9, 131.2, 130.5, 129.9, 128.8, 128.3, 127.6, 127.5, 125.9, 100.3 (d, *J* = 12.0 Hz). **^{19}F NMR** (376 MHz, Chloroform-*d*) δ -130.57 (d, *J* = 8.7 Hz). **HRMS (EI)** calculated for $C_{15}H_{10}FNO$ $[M]^+$ 239.0746; found: 239.0743.

2-(3,4-dimethylphenyl)-5-fluorooxazole (4t):

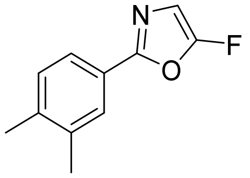

White solid 33 mg (0.17 mmol, 87%). ^{1}**H NMR** (400 MHz, Chloroform-*d*) δ 7.71 (s, 1H), 7.65 (d, *J* = 7.9 Hz, 1H), 7.20 (d, *J* = 7.9 Hz, 1H), 6.43 (d, *J* = 8.8 Hz, 1H), 2.31 (s, 3H), 2.30 (s, 3H) ^{13}**C NMR** (101 MHz, Chloroform-*d*) δ 158.4 (d, *J* = 285.2 Hz), 153.0 (d, *J* = 6.7 Hz), 139.6, 137.4, 130.2, 126.9, 124.6, 123.3, 100.4 (d, *J* = 12.4 Hz), 19.9, 19.8. ^{19}**F NMR** (376 MHz, Chloroform-*d*) δ -131.23 (d, *J* = 8.8 Hz). **HRMS (EI)** calculated for $C_{11}H_{10}FNO$ $[M]^+$ 191.0746; found: 191.0742.

2-(3,5-dichlorophenyl)-5-fluorooxazole (4u):

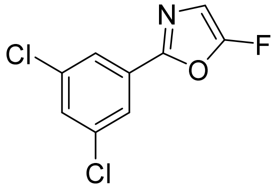

White solid 36 mg (0.15 mmol, 77%). ^{1}**H NMR** (400 MHz, Chloroform-*d*) δ 7.81 (s, 2H), 7.42 (s, 1H), 6.52 (d, *J* = 8.8 Hz, 1H). ^{13}**C NMR** (101 MHz, Chloroform-*d*) δ 158.7 (d, *J* = 288.6 Hz), 150.1 (d, *J* = 7.3 Hz), 135.9, 130.4, 129.5, 124.1, 101.6 (d, *J* = 12.3 Hz). ^{19}**F NMR** (376 MHz, Chloroform-*d*) δ -128.48 (d, *J* = 8.8 Hz). **HRMS (EI)** calculated for $C_9H_4Cl_2FNO$ $[M]^+$ 230.9654; found: 230.9649.

1-(4-bromophenyl)-2,2-difluoroaziridine (5a)

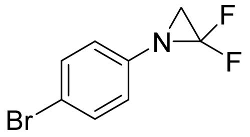

Colorless oil 32 mg (0.14 mmol, 69%). ^{1}**H NMR** (400 MHz, Chloroform-*d*) δ 7.43 (d, *J* = 8.7 Hz, 2H), 6.89 (d, *J* = 8.7 Hz, 2H), 2.86 (s, 2H) ^{13}**C NMR** (101 MHz, Chloroform-*d*) δ 143.1 (t, *J* = 2.4 Hz), 132.3, 121.9, 117.2, 106.6 (t, *J* = 282.9 Hz), 38.6 (t, *J* = 18.2 Hz). ^{19}**F NMR** (376 MHz, Chloroform-*d*) δ -119.05 (s, 2F). **HRMS (EI)** calculated for $C_8H_6BrF_2N$ $[M]^+$ 232.9652; found: 232.9649.

2,2-difluoro-1-(naphthalen-2-yl)aziridine (5b):

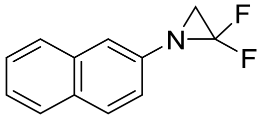

Colorless oil 27 mg (0.13 mmol, 66%). ^{1}**H NMR** (400 MHz, Chloroform-*d*) δ 7.80 (dd, *J* = 8.4, 2.1 Hz, 2H), 7.76 (d, *J* = 8.3 Hz, 1H), 7.47 (ddd, *J* = 8.2, 6.9, 1.4 Hz, 1H), 7.43 – 7.37 (m, 1H), 7.35 (d, *J* = 2.2 Hz, 1H), 7.28 – 7.19 (m, 1H), 2.97 (s, 2H). ^{13}**C NMR** (101 MHz, Chloroform-*d*) δ 141.6, 133.9, 130.8, 129.3, 127.9, 127.2, 126.8, 125.1, 120.7, 116.1, 107.1 (t, *J* = 282.4 Hz), 38.5 (t, *J* = 18.2 Hz). ^{19}**F NMR** (376 MHz, Chloroform-*d*) δ -118.76 (s, 2F). **HRMS (EI)** calculated for $C_{12}H_9F_2N$ $[M]^+$ 205.0703; found: 205.0701.

2,2-difluoro-1-tritylaziridine (5c):

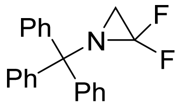

White solid 28 mg (0.09 mmol, 43%). **^{1}H NMR** (400 MHz, Chloroform-*d*) δ 7.45 (d, *J* = 7.7 Hz, 6H), 7.28 (dd, *J* = 8.4, 6.3 Hz, 6H), 7.24 – 7.19 (m, 3H), 2.19 (s, 2H). **^{13}C NMR** (101 MHz, Chloroform-*d*) δ 143.8, 129.2, 127.9, 127.3, 108.5 (t, *J* = 282.2 Hz), 73.7, 32.9 (t, *J* = 18.3 Hz). **^{19}F NMR** (376 MHz, Chloroform-*d*) δ -113.06 (s, 2F). **HRMS (EI)** calculated for $C_{21}H_{17}F_2N$ $[M]^+$ 321.1329; found: 321.1326.

1-([1,1'-biphenyl]-4-yl)-2,2-difluoroaziridine (5d):

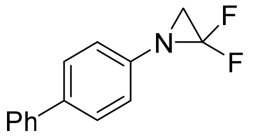

Colorless oil 20 mg (0.09 mmol, 43%). **^{1}H NMR** (400 MHz, Chloroform-*d*) δ 7.67 – 7.52 (m, 4H), 7.44 (t, *J* = 7.6 Hz, 2H), 7.37 – 7.30 (m, 1H), 7.09 (d, *J* = 8.6 Hz, 2H), 2.91 (s, 2H). **^{13}C NMR** (101 MHz, Chloroform-*d*) δ 143.2, 140.6, 137.3, 128.9, 128.0, 127.3, 127.1, 120.5, 106.9 (t, *J* = 282.4 Hz), 38.50 (t, *J* = 18.3 Hz). **^{19}F NMR** (376 MHz, Chloroform-*d*) δ -118.82 (s, 2F). **HRMS (EI)** calculated for $C_{14}H_{11}F_2N$ $[M]^+$ 231.0860; found: 231.0853.

11. Quantum chemical calculations

11.1 Computational methodology

All DFT calculation were performed with Gaussian 16 program.[3] Structure optimizations were carried out at the B3LYP level in the solution phase using the def2SVP basis set for all atoms. Solvent effect of DCM was evaluated by the self-consistent reaction field (SCRF) method using the PCM model. The vibrational frequencies were computed at the same level to check whether each optimized structure is an energy minimum (no imaginary frequency) or a transition state (one imaginary frequency) and to evaluate its zero-point vibrational energy (ZPVE) and thermal corrections at 298 K. Intrinsic reaction coordinate (IRC) were calculated to confirm the connection between the transition states and the correct reactants/products. The single point energy considering the solvent effect of DCM was obtained via calculation of the B3LYP geometries with PBE1PBE functional theory, using the def2TZVP basis set for all atoms. Solvation was evaluated by the self-consistent reaction field (SCRF) method using the SMD model. Dispersion interactions was considered both in structure optimizations and single point energy using DFT-D (gd3bj). The Gibbs free energy used for discussion in this study was calculated by adding the solution-phase Gibbs free

energy correction at the B3LYP level with the solution-phase single-point energy at the PBE1PBE level. The corresponding iodonium ions (**1**-OTf $^{-}$) were adopted when computing ADCH charge of gem-difluorovinyl iodonium reagents.

12. Supplementary References

[1] F. A. Akkerman, R. Kickbusch, D. Lentz, *Chem. Asian J.* **2008**, *3*, 719-731.

[2] F. L. Vaillant, M. D. Wodrich, J. Waser, *Chem. Sci.* **2017**, *8*, 1790-1800.

[3] Gaussian 16, Revision A.03, M. J. Frisch, G. W. Trucks, H. B. Schlegel, G. E. Scuseria, M. A. Robb, J. R. Cheeseman, G. Scalmani, V. Barone, G. A. Petersson, H. Nakatsuji, X. Li, M. Caricato, A. V. Marenich, J. Bloino, B. G. Janesko, R. Gomperts, B. Mennucci, H. P. Hratchian, J. V. Ortiz, A. F. Izmaylov, J. L. Sonnenberg, D. Williams-Young, F. Ding, F. Lipparini, F. Egidi, J. Goings, B. Peng, A. Petrone, T. Henderson, D. Ranasinghe, V. G. Zakrzewski, J. Gao, N. Rega, G. Zheng, W. Liang, M. Hada, M. Ehara, K. Toyota, R. Fukuda, J. Hasegawa, M. Ishida, T. Nakajima, Y. Honda, O. Kitao, H. Nakai, T. Vreven, K. Throssell, J. A. Montgomery, Jr., J. E. Peralta, F. Ogliaro, M. J. Bearpark, J. J. Heyd, E. N. Brothers, K. N. Kudin, V. N. Staroverov, T. A. Keith, R. Kobayashi, J. Normand, K. Raghavachari, A. P. Rendell, J. C. Burant, S. S. Iyengar, J. Tomasi, M. Cossi, J. M. Millam, M. Klene, C. Adamo, R. Cammi, J. W. Ochterski, R. L. Martin, K. Morokuma, O. Farkas, J. B. Foresman, and D. J. Fox, Gaussian, Inc., Wallingford CT, 2016.
