# Supplementary material for: Diverse reactivity of the gem-difluorovinyl iodonium salt for direct incorporation of the difluoroethylene group into N- and O-nucleophiles
Source: Commun Chem. 2022 Dec 3;5:167. doi: 10.1038/s42004-022-00772-7 (PMC9814539; doi:10.1038/s42004-022-00772-7)
Supplement: Supplementary file 2 — Description of Additional Supplementary Files [file 42004_2022_772_MOESM2_ESM.pdf]

Description of Additional Supplementary Files

File name: Supplementary Data 1

Description: 1a.cif

File name: Supplementary Data 2

Description: 3a.cif

File name: Supplementary Data 3

Description: 4j.cif

File name: Supplementary Data 4

Description: Cartesian coordinates and energies of all computed structures

File name: Supplementary Data 5

Description: Copies of NMR spectra for compounds
