# Supplementary material for: Diverse reactivity of the gem-difluorovinyl iodonium salt for direct incorporation of the difluoroethylene group into N- and O-nucleophiles
Source: Commun Chem. 2022 Dec 3;5:167. doi: 10.1038/s42004-022-00772-7 (PMC9814539; doi:10.1038/s42004-022-00772-7)
Supplement: Supplementary file 6 — Supplementary Data 4 [file 42004_2022_772_MOESM6_ESM.docx]

**Cartesian coordinates and energies of all computed structures**

**1a**-OTf ^-^

B3LYP/def2SVP thermal correction to Gibbs Free Energy: 0.078821 a.u.

PBE1PBE/def2TZVP SCF energy in solution: -805.268823666 a.u.

Charge = 1 Multiplicity = 1

C 3.35374200 0.23352500 1.12412000

C 2.13538700 -0.44655600 1.03135300

C 1.31299900 -0.14021000 -0.05226200

C 1.63628000 0.79014800 -1.03971900

C 2.86156300 1.45308300 -0.91895900

C 3.71262800 1.17635900 0.15566200

H 4.02140800 0.01912900 1.96090100

H 1.84858700 -1.18331100 1.78213000

H 0.96855400 1.00236200 -1.87512800

H 3.14564800 2.18860700 -1.67409300

H 4.66686200 1.70024600 0.23830400

I -0.56181900 -1.16245100 -0.21274200

C -1.75738300 0.14182200 0.89104600

C -2.35737500 1.18291100 0.30241100

H -1.86384300 -0.07299000 1.95281600

F -3.11591700 2.01561500 0.94919400

F -2.26162500 1.49654000 -0.95713900

**1b**-OTf ^–^

Charge = 1 Multiplicity = 1

C 2.20659200 -0.85474700 0.92550700

C 0.85355300 -1.19860300 0.87256700

C 0.07134000 -0.58716900 -0.10453900

C 0.56134300 0.33656300 -1.02683800

C 1.91482900 0.66880900 -0.94963900

C 2.72948900 0.07309200 0.01993600

H 2.84738700 -1.30936300 1.68169900

H 0.43989600 -1.91841300 1.57907800

H -0.07647100 0.79471700 -1.78258800

H 2.32899000 1.39667900 -1.64859400

I -2.00708500 -1.10002400 -0.18882600

C -2.78604100 0.47531400 0.92846700

C -3.21189900 1.59265500 0.32657600

H -2.84356500 0.32846600 2.00550700

F -3.72404800 2.58954600 0.98074600

F -3.16802500 1.82463100 -0.95277900

C 4.20404200 0.40713600 0.05550600

F 4.72791900 0.21485100 1.27510200

F 4.89361500 -0.36514800 -0.80260600

F 4.42829500 1.68399700 -0.29074700

**1c**-OTf ^–^

Charge = 1 Multiplicity = 1

C 2.06940100 -0.99158400 0.11503400

C 0.75398300 -1.34466400 -0.19795700

C -0.13170200 -0.31499400 -0.50623100

C 0.22729600 1.02946600 -0.52711200

C 1.55058100 1.34972700 -0.20605700

C 2.46963100 0.34733700 0.10961400

H 0.44949600 -2.39091600 -0.20195700

H -0.48211500 1.81483600 -0.78682100

H 3.50288800 0.60876100 0.33926400

I -2.15367500 -0.82782000 -0.98419000

C -2.96871400 -0.71295100 0.92786600

C -3.47624200 0.43759500 1.38929000

H -2.96736100 -1.63127700 1.51237500

F -4.00603900 0.54332400 2.56829600

F -3.50131800 1.56531100 0.74352800

C 1.96988400 2.80408700 -0.16824600

C 3.06365500 -2.06699400 0.49811200

F 1.73293400 3.34042500 1.04028100

F 3.27709800 2.94495300 -0.42244600

F 1.29079700 3.53081300 -1.06818700

F 4.29081700 -1.77786900 0.04397400

F 2.70936100 -3.26259100 0.00552200

F 3.14804400 -2.18982400 1.83273800

**AgOTf**

B3LYP/def2SVP thermal correction to Gibbs Free Energy: -0.009788 a.u.

PBE1PBE/def2TZVP SCF energy in solution: -1108.09655622 a.u.

Charge = 0 Multiplicity = 1

Ag 2.04835600 -0.20248900 -0.00000200

C -1.97853000 -0.57821600 -0.00000300

S -0.73395600 0.81072500 0.00001400

F -2.75043900 -0.50779200 -1.08327900

F -1.34818000 -1.75672300 0.00001300

F -2.75053100 -0.50782800 1.08321500

O 0.07741300 0.54121600 -1.23230300

O -1.53170700 2.03822300 -0.00006500

O 0.07730300 0.54128200 1.23241100

**IM1**

B3LYP/def2SVP thermal correction to Gibbs Free Energy: 0.091340 a.u.

PBE1PBE/def2TZVP SCF energy in solution: -1913.38031308 a.u.

Charge = 1 Multiplicity = 1

C -0.23339700 3.09324000 -0.94226400

C -1.33528300 2.23564400 -1.00854600

C -1.70598500 1.58326000 0.17069300

C -1.06860200 1.76119600 1.39949700

C 0.03427200 2.61848100 1.42511000

C 0.45044000 3.27426000 0.26347500

H 0.09479500 3.60777100 -1.84697000

H -1.86802900 2.07983400 -1.94712400

H -1.38508600 1.22876600 2.29666800

H 0.58412500 2.74910000 2.35795700

H 1.33070300 3.91643700 0.29522200

C -2.11547200 -1.50728900 -0.48263300

C -1.69040800 -2.36866200 0.48114300

H -2.22349600 -1.83947600 -1.51757800

F -1.16598000 -3.51616200 0.20951300

F -1.74604100 -2.13983400 1.75025400

I -3.29901300 0.16110900 0.06068200

Ag 0.12528900 -0.61003400 -0.87017000

C 4.11754100 -0.65854000 0.11617700

S 2.66285600 0.50211500 0.25011100

F 4.71573900 -0.77601600 1.29904500

F 3.70022800 -1.86501200 -0.27651300

F 4.99621500 -0.19895500 -0.77073700

O 1.75127000 -0.16670700 1.21491200

O 3.23636400 1.79252400 0.63748500

O 2.08740400 0.45303800 -1.15215700

**Amide** (1-naphthamide)

B3LYP/def2SVP thermal correction to Gibbs Free Energy: 0.139267 a.u.

PBE1PBE/def2TZVP SCF energy in solution: -554.197333967 a.u.

Charge = 0 Multiplicity = 1

C 2.01364600 -1.95406100 0.13006900

C 0.68917300 -1.57345700 0.06366700

C 0.32535300 -0.19753200 -0.00715800

C 1.37695800 0.78292100 -0.00846100

C 2.73200900 0.35364000 0.05883200

C 3.04761800 -0.98575700 0.12661300

H 2.26872900 -3.01516400 0.18397500

H -0.09823900 -2.32433800 0.04816400

C -1.03544400 0.26107700 -0.05334200

C 1.05077900 2.16558600 -0.07547600

H 3.51961800 1.11132400 0.05751000

H 4.09144200 -1.30395600 0.17823900

C -0.26179500 2.57749200 -0.12933600

C -1.30250500 1.62024500 -0.11180700

H 1.86368000 2.89599300 -0.08597400

H -0.50790800 3.63976100 -0.18949000

H -2.33726600 1.96362000 -0.17200200

C -2.18432100 -0.71434300 -0.08447800

O -2.15544700 -1.75779200 -0.72973100

N -3.27572000 -0.34191500 0.63384700

H -4.07855100 -0.96132400 0.64463400

H -3.25671700 0.43495500 1.28113500

**TS1**

B3LYP/def2SVP thermal correction to Gibbs Free Energy: 0.249650 a.u.

PBE1PBE/def2TZVP SCF energy in solution: -2467.58671376 a.u.

Charge = 1 Multiplicity = 1

C -4.40305100 -2.48816700 -0.72446400

C -3.05842500 -2.85954900 -0.81127400

C -2.23525300 -2.55341200 0.27452000

C -2.68942700 -1.93243000 1.43864500

C -4.03638800 -1.56586500 1.49131500

C -4.88377900 -1.83302700 0.41326000

H -5.06954800 -2.69930700 -1.56289500

H -2.67103900 -3.34716200 -1.70637400

H -2.01707200 -1.69955000 2.26445200

H -4.41056000 -1.03562300 2.36745700

H -5.92420400 -1.50918200 0.45452900

C 0.35184600 -0.98422000 -0.77217000

C 1.26408600 -0.26861200 0.04505200

H 0.72530500 -1.15521500 -1.78579800

F 1.93267500 0.70900200 -0.47925300

F 1.05948700 -0.09967600 1.31447900

I -0.13963200 -2.89787000 0.08392600

Ag -1.47354100 0.21599600 -0.95647200

C -3.85835600 3.52719400 -0.03663100

S -3.91404400 1.68542000 0.25650900

F -4.22019700 4.17576800 1.06912800

F -2.61762600 3.89970200 -0.36441900

F -4.68186000 3.86875800 -1.02569000

O -2.93755300 1.45663700 1.34042500

O -5.32310500 1.38631300 0.52740900

O -3.42580300 1.15990400 -1.08426800

C 6.69925200 1.22457300 -2.06637500

C 5.81864200 0.40560500 -1.38980200

C 5.42357700 0.70226500 -0.05430400

C 5.94148900 1.89439300 0.55915900

C 6.85217900 2.71163500 -0.16606900

C 7.22946200 2.38370900 -1.44984400

H 6.98564700 0.97861500 -3.09122600

H 5.41594300 -0.47209100 -1.89536000

C 4.49984400 -0.09139100 0.70575200

C 5.52613200 2.24758100 1.87193500

H 7.24157400 3.61175700 0.31512200

H 7.92797200 3.01910000 -1.99819300

C 4.61840700 1.47536700 2.56410200

C 4.09671800 0.30563100 1.97224500

H 5.93762000 3.15355800 2.32290800

H 4.30217200 1.75721000 3.56992800

H 3.38627200 -0.30911600 2.52653100

C 3.93721400 -1.37166800 0.20086800

O 2.69827700 -1.61581500 0.24364800

N 4.76703600 -2.30122700 -0.26042000

H 4.39756500 -3.18481600 -0.60057900

H 5.77288000 -2.18696200 -0.19702800

**IM2**

B3LYP/def2SVP thermal correction to Gibbs Free Energy: 0.254251 a.u.

PBE1PBE/def2TZVP SCF energy in solution: -2467.60093698 a.u.

Charge = 1 Multiplicity = 1

C -4.63683600 -2.13769800 0.33596500

C -3.45132600 -2.76178100 -0.06551000

C -2.28980900 -2.45478400 0.64356400

C -2.25880900 -1.58533100 1.73431300

C -3.45571300 -0.96646100 2.10313100

C -4.63485000 -1.23464400 1.40265100

H -5.55991800 -2.34657500 -0.20841600

H -3.43987900 -3.44107900 -0.91840300

H -1.33023500 -1.35475900 2.25641200

H -3.45255500 -0.24373400 2.91944300

H -5.55396400 -0.71715400 1.67901400

C 0.15937800 -1.30729400 -1.08433000

C 1.34837000 -0.74969200 -0.36526200

H 0.46168900 -1.64208800 -2.08483600

F 1.78537000 0.35199500 -0.99909100

F 1.08849600 -0.42434700 0.91200800

I -0.43157600 -3.18621200 -0.10631700

Ag -1.49878200 0.04331500 -1.08754000

C -3.27433700 3.81569000 -0.28603000

S -3.42313400 2.06144400 0.33142500

F -3.35608100 4.66700000 0.73638800

F -2.09725500 3.99083900 -0.89356200

F -4.24684600 4.09266600 -1.15362000

O -2.27678900 1.89850600 1.24549200

O -4.77113600 1.97754200 0.90323500

O -3.26579800 1.28429100 -0.96481900

C 6.46308500 1.80668500 -1.67124100

C 5.55903900 0.83835600 -1.28126900

C 5.14760500 0.73354100 0.07441300

C 5.66082700 1.68573900 1.01845900

C 6.59487000 2.66540700 0.58363500

C 6.99596700 2.72200000 -0.73328100

H 6.76258400 1.87115400 -2.71928700

H 5.14981000 0.16684200 -2.03617800

C 4.19346800 -0.23076900 0.55966100

C 5.21629600 1.64883800 2.36748200

H 6.98013200 3.37949200 1.31464600

H 7.71204100 3.47859700 -1.05966400

C 4.28263900 0.72625500 2.79316900

C 3.76148000 -0.21218600 1.88412600

H 5.62659800 2.37765700 3.07010300

H 3.95004300 0.71011800 3.83190300

H 3.04711900 -0.95701200 2.23323500

C 3.72064800 -1.32669800 -0.27940000

O 2.45018200 -1.69779200 -0.35496100

N 4.53545000 -2.07362200 -0.97397900

H 4.17488800 -2.80411300 -1.58687900

H 5.54314100 -1.96643500 -0.88303200

**OTf** ^-^

B3LYP/def2SVP thermal correction to Gibbs Free Energy: -0.005128 a.u.

PBE1PBE/def2TZVP SCF energy in solution: -961.234044356 a.u.

Charge = -1 Multiplicity = 1

C -0.95191700 0.00006700 -0.00003600

S 0.91894600 0.00001700 -0.00000300

F -1.43802600 0.89563400 0.87280100

F -1.43821700 0.30808700 -1.21202200

F -1.43815900 -1.20360200 0.33913200

O 1.24348000 1.38773400 -0.39843000

O 1.24296600 -0.34891300 1.40105000

O 1.24330000 -1.03904000 -1.00248600

**TfOH**

B3LYP/def2SVP thermal correction to Gibbs Free Energy: 0.005065 a.u.

PBE1PBE/def2TZVP SCF energy in solution: -961.662121945 a.u.

Charge = 0 Multiplicity = 1

C 1.00913600 0.00627000 -0.00255200

S -0.85961900 -0.14164000 0.06687100

F 1.42053400 -0.11970500 -1.25365000

F 1.37186500 1.19132700 0.47248100

F 1.53431400 -0.95399800 0.74320400

O -1.22767700 -1.28865200 -0.73658000

O -1.24801900 0.02218600 1.45787200

O -1.26002900 1.16990200 -0.78593600

H -1.35553000 1.94251200 -0.19578100

**IM3**

B3LYP/def2SVP thermal correction to Gibbs Free Energy: 0.239826 a.u.

PBE1PBE/def2TZVP SCF energy in solution: -2467.15560140 a.u.

Charge = 0 Multiplicity = 1

C -4.41342800 -2.27136600 0.74304300

C -3.26861400 -2.88360000 0.22186800

C -2.03365200 -2.49857000 0.74316000

C -1.89258400 -1.55591500 1.76269100

C -3.05136500 -0.95113200 2.25573200

C -4.30343200 -1.30090200 1.74266000

H -5.39260000 -2.54252800 0.34298700

H -3.34533100 -3.61551200 -0.58294000

H -0.91023600 -1.25953100 2.13081400

H -2.96456000 -0.17400700 3.01537400

H -5.19621200 -0.79389000 2.11014400

C 0.19907500 -1.31634700 -1.24368400

C 1.47089900 -0.78145400 -0.62604300

H 0.43093700 -1.68566000 -2.25158400

F 1.84566400 0.32132400 -1.33259600

F 1.25156800 -0.35769500 0.65374300

I -0.25965300 -3.21491600 -0.19507300

Ag -1.47143500 -0.01113700 -1.10813500

C -3.34250100 3.72423600 -0.30130100

S -3.35789600 1.99908100 0.40882600

F -3.38620200 4.62329600 0.68275700

F -2.22774000 3.92241300 -1.01002200

F -4.39331400 3.91345800 -1.09974100

O -2.13743600 1.93364800 1.23337900

O -4.65127900 1.89100500 1.09375300

O -3.27322700 1.16499200 -0.85633900

C 5.75763600 2.49075400 -1.48092600

C 5.12282600 1.29683700 -1.21009400

C 4.86618600 0.89462400 0.13159500

C 5.26723300 1.76962600 1.19752400

C 5.92586800 2.99082100 0.88111000

C 6.17017200 3.34476800 -0.42782800

H 5.94035500 2.78375500 -2.51728100

H 4.80258200 0.65720900 -2.03283400

C 4.19646300 -0.32606100 0.47324900

C 4.99160300 1.40492100 2.54348200

H 6.23004100 3.64699200 1.70025900

H 6.67434400 4.28581800 -0.65861800

C 4.33263500 0.23053600 2.83796400

C 3.92619800 -0.63263200 1.79644800

H 5.30706400 2.07887400 3.34365900

H 4.12034500 -0.03945100 3.87440600

H 3.39761200 -1.55693000 2.03652900

C 3.83000900 -1.30916200 -0.58284000

O 2.49486600 -1.70930900 -0.64675900

N 4.60041800 -1.88931900 -1.39827200

H 5.56762200 -1.59898900 -1.22942800

**IM3'**

B3LYP/def2SVP thermal correction to Gibbs Free Energy: 0.244807 a.u.

PBE1PBE/def2TZVP SCF energy in solution: -2467.16367019 a.u.

Charge = 0 Multiplicity = 1

C -4.64592400 1.59732100 -0.68694700

C -4.42283900 0.22407600 -0.83737800

C -3.74289700 -0.43837300 0.18437700

C -3.30231900 0.19651100 1.34701000

C -3.52481300 1.57108600 1.46370500

C -4.18351700 2.26911500 0.44760800

H -5.16198800 2.14334700 -1.47933200

H -4.74586700 -0.29669900 -1.73948000

H -2.75792600 -0.34610500 2.11902100

H -3.14706500 2.10046100 2.33943600

H -4.31726800 3.34802300 0.53429400

C -0.97870900 -1.92348200 -0.41693100

C -0.24276600 -2.36061600 0.83594100

H -0.69865300 -2.57153300 -1.25259200

F -0.89489800 -1.96178100 1.96027200

F -0.12843800 -3.71485300 0.94667500

I -3.12650400 -2.45545100 -0.14705600

Ag -0.84878100 0.15710700 -0.82502100

C 1.06714200 3.91230400 -0.27918000

S -0.52952800 3.06935200 0.20684300

F 1.67533000 4.40245300 0.80392800

F 1.89141600 3.04145700 -0.86858200

F 0.82937200 4.91177400 -1.12604300

O -0.15066000 2.17524400 1.31997600

O -1.45543000 4.16617700 0.50749500

O -0.86023900 2.32012500 -1.06964600

C 6.17037300 -2.75391900 -0.68070500

C 4.83839800 -2.48256900 -0.44230300

C 4.42001200 -1.19970800 0.01433900

C 5.42888000 -0.20458000 0.25388800

C 6.79163500 -0.51618600 -0.00986000

C 7.15819000 -1.76069500 -0.47451300

H 6.46573000 -3.74789100 -1.02439500

H 4.10042400 -3.27159100 -0.58648000

C 3.05187800 -0.85454300 0.28512000

C 5.05289400 1.06892800 0.76342500

H 7.54616300 0.25328300 0.17140900

H 8.20803700 -1.98826500 -0.67210300

C 3.73599300 1.35677800 1.04408100

C 2.73319500 0.38894000 0.81096900

H 5.83308500 1.81498700 0.93322300

H 3.45085000 2.33538000 1.43345700

H 1.69395000 0.64714100 1.01834200

C 1.93869300 -1.78459900 -0.03900700

O 1.01574500 -1.79031200 0.97409900

N 1.75488000 -2.48087700 -1.08284100

H 2.50488400 -2.32523100 -1.75790700

**TS2**

B3LYP/def2SVP thermal correction to Gibbs Free Energy: 0.243294 a.u.

PBE1PBE/def2TZVP SCF energy in solution: -2467.15039568 a.u.

Charge = 0 Multiplicity = 1

C -4.33699300 2.05682000 -0.83726200

C -4.31205300 0.66121600 -0.93332900

C -3.76467400 -0.06273600 0.12776400

C -3.26325600 0.55296700 1.27670700

C -3.28759500 1.94885800 1.34332300

C -3.81413200 2.69835600 0.28846600

H -4.74887300 2.64105800 -1.66292400

H -4.69040100 0.15936700 -1.82459600

H -2.82288500 -0.03261000 2.08272100

H -2.85583200 2.44924600 2.21105200

H -3.79275700 3.78774600 0.33657000

C -0.96750500 -1.89066900 -0.45292400

C -0.41995300 -2.34194000 0.89984200

H -1.00281500 -2.70178700 -1.17937200

F -1.16292000 -1.88544500 1.93388000

F -0.37849900 -3.69217400 1.03599200

I -3.52119800 -2.16755000 -0.08822800

Ag -0.81681200 0.15523900 -0.88881700

C 1.57481200 3.51316900 -0.27979800

S -0.15815400 2.99210600 0.18931500

F 2.23437700 3.93487100 0.80110600

F 2.24245600 2.48327800 -0.80878900

F 1.54066200 4.50011400 -1.17319400

O 0.03407500 2.05623300 1.31520300

O -0.86509000 4.24680400 0.46416200

O -0.61133800 2.30447300 -1.08562300

C 5.73213300 -2.93944900 -1.14541800

C 4.46754800 -2.60040100 -0.70858900

C 4.24221000 -1.39560900 0.01510900

C 5.36947400 -0.55433300 0.30592600

C 6.65793000 -0.93282700 -0.16270000

C 6.83813300 -2.09642500 -0.87918800

H 5.88198600 -3.87121600 -1.69545000

H 3.63488400 -3.27591400 -0.90657000

C 2.95503200 -0.98525200 0.49946900

C 5.17989000 0.63731700 1.05941000

H 7.50633000 -0.28183200 0.06171800

H 7.83242200 -2.37645800 -1.23393700

C 3.93324700 0.99025000 1.52666100

C 2.81619600 0.16993000 1.25452400

H 6.04696900 1.27151300 1.25956500

H 3.79100400 1.91422900 2.08959100

H 1.82703300 0.48351700 1.58999600

C 1.72223000 -1.71504700 0.13574400

O 0.85887400 -1.83783800 1.17429200

N 1.34335200 -2.14783800 -0.99858700

H 1.98076100 -1.93014700 -1.76444300

**PhI**

B3LYP/def2SVP thermal correction to Gibbs Free Energy: 0.058372 a.u.

PBE1PBE/def2TZVP SCF energy in solution: -529.187568525 a.u.

Charge = 0 Multiplicity = 1

C -2.65334300 -1.20958100 0.00000000

C -1.25440500 -1.21839400 0.00000100

C -0.56887400 -0.00000600 0.00000000

C -1.25440200 1.21839200 0.00000000

C -2.65333200 1.20958700 0.00000100

C -3.35448400 0.00000200 0.00000000

H -3.19380700 -2.15924800 0.00000100

H -0.71034800 -2.16424800 0.00000000

H -0.71032900 2.16423700 0.00000000

H -3.19380100 2.15925100 0.00000100

H -4.44673600 0.00001100 0.00000000

I 1.56015200 0.00000000 0.00000000

**IM4**

B3LYP/def2SVP thermal correction to Gibbs Free Energy: 0.163927 a.u.

PBE1PBE/def2TZVP SCF energy in solution: -1938.03127778 a.u.

Charge = 0 Multiplicity = 1

C 0.33130100 -1.87569200 -0.34535800

C 0.46099900 -2.18751500 1.13014700

H 0.35992400 -2.80016700 -0.93653400

F -0.57966600 -1.83025000 1.90603200

F 0.69714000 -3.48433500 1.40989500

Ag -1.43503800 -0.80125500 -0.87460300

C -3.93131800 1.80615400 0.58800800

S -4.32986300 0.36359600 -0.53010000

F -4.87526500 1.93809300 1.52021800

F -2.75610100 1.61637300 1.20036000

F -3.86158700 2.93855300 -0.11302400

O -4.33666100 -0.80215800 0.36974900

O -5.58640400 0.73938000 -1.18376200

O -3.14468200 0.38005100 -1.47944200

C 5.34083900 0.07595000 -2.39355700

C 4.37975500 -0.29120000 -1.47153300

C 4.27966100 0.36662600 -0.21587000

C 5.24906100 1.38094100 0.09314500

C 6.21902100 1.74134800 -0.88206100

C 6.25893700 1.11265300 -2.10767800

H 5.39909800 -0.45156700 -3.34793000

H 3.72753500 -1.13048500 -1.71005600

C 3.29803200 0.05446100 0.79196800

C 5.24516000 1.99421700 1.37548500

H 6.94172800 2.52204000 -0.63439200

H 7.00833300 1.39585000 -2.84941900

C 4.33364700 1.62447900 2.34235700

C 3.35717900 0.65460900 2.04873100

H 5.99101200 2.76384300 1.58714300

H 4.34861000 2.09214600 3.32804100

H 2.61142700 0.39279100 2.79968800

C 2.17775300 -0.83487400 0.55216100

O 1.58968900 -1.43747900 1.58786300

N 1.54006300 -1.09650100 -0.55853700

H 1.76008800 -0.64469600 -1.44113600

**TS3**

B3LYP/def2SVP thermal correction to Gibbs Free Energy: 0.162384 a.u.

PBE1PBE/def2TZVP SCF energy in solution: -1938.00585686 a.u.

Charge = 0 Multiplicity = 1

C -0.36731500 1.60621100 -0.94415900

C -0.53337000 2.83402200 -0.24453600

H -0.50880300 1.78053100 -2.02108700

F -0.33097700 2.95763200 1.03592400

F -0.41278000 3.97136500 -0.84659500

Ag 1.68431100 0.96381100 -0.73274100

C 3.84062200 -2.16452800 0.27626800

S 4.11515800 -0.36623300 0.69852900

F 4.08574100 -2.92881800 1.34023500

F 2.57262400 -2.36376700 -0.10527400

F 4.64400100 -2.54259400 -0.71750900

O 3.14632700 -0.09551900 1.77579900

O 5.54269500 -0.26736000 1.01042900

O 3.74506800 0.30858200 -0.61181500

C -3.91949400 -2.48635200 -1.89508000

C -3.47982900 -1.29272100 -1.35905200

C -4.03983800 -0.77703600 -0.15559800

C -5.11362100 -1.51197300 0.45656800

C -5.53567400 -2.74263500 -0.11825200

C -4.94708600 -3.22812000 -1.26539200

H -3.47496000 -2.85619200 -2.82164200

H -2.70975400 -0.73180900 -1.88720900

C -3.63874600 0.45728200 0.46307100

C -5.75505600 -0.99128400 1.61347600

H -6.34662600 -3.29196200 0.36565000

H -5.28027700 -4.17312500 -1.69941800

C -5.37921000 0.22039700 2.15208500

C -4.32034100 0.94667500 1.56930000

H -6.56506500 -1.56945400 2.06446700

H -5.88592400 0.61776600 3.03335300

H -4.00419400 1.89588700 2.00407900

C -2.48961600 1.26627300 0.00464900

O -2.51085000 2.52062000 0.04285400

N -1.34000700 0.68716200 -0.40482900

H -1.19145100 -0.31337900 -0.36127700

**3a**

B3LYP/def2SVP thermal correction to Gibbs Free Energy: 0.151415 a.u.

PBE1PBE/def2TZVP SCF energy in solution: -829.906468312 a.u.

Charge = 0 Multiplicity = 1

C -3.20110200 -2.12293300 -0.39961600

C -1.95938700 -1.60710200 -0.09013200

C -1.76813000 -0.20386000 0.06357800

C -2.90348000 0.65986800 -0.11409700

C -4.16955100 0.09238900 -0.42986100

C -4.31906200 -1.27004400 -0.56985200

H -3.32406300 -3.20263500 -0.51291200

H -1.11076700 -2.27254800 0.05488200

C -0.49660200 0.39598500 0.36231300

C -2.74728500 2.06654100 0.02479300

H -5.02313000 0.76190600 -0.56160600

H -5.29569600 -1.69496000 -0.81264100

C -1.51900200 2.61418600 0.32017700

C -0.39407700 1.77406700 0.48386400

H -3.62354000 2.70648500 -0.10500100

H -1.40577600 3.69404200 0.43538700

H 0.56601300 2.22592200 0.74217100

C 0.72009600 -0.45223400 0.58728600

O 0.70855400 -1.51670400 1.19388400

N 1.88209100 0.06462100 0.06485200

H 1.83787400 0.90085900 -0.50661700

C 3.10870400 -0.57698300 0.22545900

H 3.09497700 -1.49587700 0.80813600

C 4.24697900 -0.11909800 -0.29360000

F 5.42024200 -0.70678300 -0.15949400

F 4.34114000 0.99049300 -1.01480600
