# Supplementary material for: Diverse reactivity of the gem-difluorovinyl iodonium salt for direct incorporation of the difluoroethylene group into N- and O-nucleophiles
Source: Commun Chem. 2022 Dec 3;5:167. doi: 10.1038/s42004-022-00772-7 (PMC9814539; doi:10.1038/s42004-022-00772-7)
Supplement: Supplementary file 7 — Supplementary Data 5 [file 42004_2022_772_MOESM7_ESM.docx]

**Copies of NMR spectra for compounds**

**(2,2-difluorovinyl)(phenyl)-λ^3^-iodanyl trifluoromethanesulfonate (1a) ^1^H NMR**

**(2,2-difluorovinyl)(phenyl)-λ^3^-iodanyl trifluoromethanesulfonate (1a) ^13^CNMR**

**(2,2-difluorovinyl)(phenyl)-λ^3^-iodanyl trifluoromethanesulfonate (1a) ^19^F NMR**

**(2,2-difluorovinyl)(4-(trifluoromethyl)phenyl)-λ^3^-iodanyl trifluoromethanesulfonate (1b) ^1^H NMR**

**(2,2-difluorovinyl)(4-(trifluoromethyl)phenyl)-λ^3^-iodanyl trifluoromethanesulfonate (1b) ^13^C NMR**

**(2,2-difluorovinyl)(4-(trifluoromethyl)phenyl)-λ^3^-iodanyl trifluoromethanesulfonate (1b) ^19^F NMR**

**(3,5-bis(trifluoromethyl)phenyl)(2,2-difluorovinyl)-λ3-iodanyl trifluoromethanesulfonate (1c) ^1^H NMR**

**(3,5-bis(trifluoromethyl)phenyl)(2,2-difluorovinyl)-λ3-iodanyl trifluoromethanesulfonate (1c) ^13^C NMR**

**(3,5-bis(trifluoromethyl)phenyl)(2,2-difluorovinyl)-λ3-iodanyl trifluoromethanesulfonate (1c) ^19^F NMR**

**2,2-difluorovinyl [1,1'-biphenyl]-2-carboxylate (2a) ^1^H NMR**

**2,2-difluorovinyl [1,1'-biphenyl]-2-carboxylate (2a) ^13^C NMR**

**2,2-difluorovinyl [1,1'-biphenyl]-2-carboxylate (2a) ^19^F NMR**

**2,2-difluorovinyl 2-bromobenzoate (2b) ^1^H NMR**

**2,2-difluorovinyl 2-bromobenzoate (2b) ^13^C NMR**

**2,2-difluorovinyl 2-bromobenzoate (2b) ^19^F NMR**

**2,2-difluorovinyl 2-bromo-5-methylbenzoate (2c) ^1^H NMR**

**2,2-difluorovinyl 2-bromo-5-methylbenzoate (2c) ^13^C NMR**

**2,2-difluorovinyl 2-bromo-5-methylbenzoate (2c) ^19^F NMR**

**2,2-difluorovinyl 4-acetylbenzoate (2d) ^1^H NMR**

**2,2-difluorovinyl 4-acetylbenzoate (2d) ^13^C NMR**

**2,2-difluorovinyl 4-acetylbenzoate (2d) ^19^F NMR**

**2,2-difluorovinyl 4-iodobenzoatee (2e) ^1^H NMR**

**2,2-difluorovinyl 4-iodobenzoatee (2e) ^13^C NMR**

**2,2-difluorovinyl 4-iodobenzoatee (2e) ^19^F NMR**

**2,2-difluorovinyl 4-fluoro-2-nitrobenzoate (2f) ^1^H NMR**

**2,2-difluorovinyl 4-fluoro-2-nitrobenzoate (2f) ^13^C NMR**

**2,2-difluorovinyl 4-fluoro-2-nitrobenzoate (2f) ^19^F NMR**

**2,2-difluorovinyl 4-methoxybenzoate (2g) ^1^H NMR**

**2,2-difluorovinyl 4-methoxybenzoate (2g) ^13^C NMR**

**2,2-difluorovinyl 4-methoxybenzoate (2g) ^19^F NMR**

**2,2-difluorovinyl cinnamate (2h) ^1^H NMR**

**2,2-difluorovinyl cinnamate (2h) ^13^C NMR**

**2,2-difluorovinyl cinnamate (2h) ^19^F NMR**

**2,2-difluorovinyl 3,5-dichlorobenzoate (2i) ^1^H NMR**

**2,2-difluorovinyl 3,5-dichlorobenzoate (2i) ^13^C NMR**

**2,2-difluorovinyl 3,5-dichlorobenzoate (2i) ^19^F NMR**

**2,2-difluorovinyl 1-naphthoate (2j) ^1^H NMR**

**2,2-difluorovinyl 1-naphthoate (2j) ^13^C NMR**

**2,2-difluorovinyl 1-naphthoate (2j) ^19^F NMR**

**2,2-difluorovinyl 2-(2-fluoro-[1,1'-biphenyl]-4-yl)propanoate (2k) ^1^H NMR**

**2,2-difluorovinyl 2-(2-fluoro-[1,1'-biphenyl]-4-yl)propanoate (2k) ^13^C NMR**

**2,2-difluorovinyl 2-(2-fluoro-[1,1'-biphenyl]-4-yl)propanoate (2k) ^19^F NMR**

**bis(2,2-difluorovinyl) terephthalate (2l) ^1^H NMR**

**bis(2,2-difluorovinyl) terephthalate (2l) ^13^C NMR**

**bis(2,2-difluorovinyl) terephthalate (2l) ^19^F NMR**

**N-(2,2-difluorovinyl)-1-naphthamide (3a) ^1^H NMR**

**N-(2,2-difluorovinyl)-1-naphthamide (3a) ^13^C NMR**

**N-(2,2-difluorovinyl)-1-naphthamide (3a) ^19^F NMR**

**N-(2,2-difluorovinyl)-2-naphthamide (3b) ^1^H NMR**

**N-(2,2-difluorovinyl)-2-naphthamide (3b) ^13^C NMR**

**N-(2,2-difluorovinyl)-2-naphthamide (3b) ^19^F NMR**

**4-(tert-butyl)-N-(2,2-difluorovinyl)benzamide (3c) ^1^H NMR**

**4-(tert-butyl)-N-(2,2-difluorovinyl)benzamide (3c) ^13^C NMR**

**4-(tert-butyl)-N-(2,2-difluorovinyl)benzamide (3c) ^19^F NMR**

**N-(2,2-difluorovinyl)-4-methylbenzamide (3d) ^1^H NMR**

**N-(2,2-difluorovinyl)-4-methylbenzamide (3d) ^13^C NMR**

**N-(2,2-difluorovinyl)-4-methylbenzamide (3d) ^19^F NMR**

**2-chloro-N-(2,2-difluorovinyl)benzamide (3e) ^1^H NMR**

**2-chloro-N-(2,2-difluorovinyl)benzamide (3e) ^13^C NMR**

**2-chloro-N-(2,2-difluorovinyl)benzamide (3e) ^19^F NMR**

**3-chloro-N-(2,2-difluorovinyl)benzamide (3f) ^1^H NMR**

**3-chloro-N-(2,2-difluorovinyl)benzamide (3f) ^13^C NMR**

**3-chloro-N-(2,2-difluorovinyl)benzamide (3f) ^19^F NMR**

**4-chloro-N-(2,2-difluorovinyl)benzamide (3g) ^1^H NMR**

**4-chloro-N-(2,2-difluorovinyl)benzamide (3g) ^13^C NMR**

**4-chloro-N-(2,2-difluorovinyl)benzamide (3g) ^19^F NMR**

**N-(2,2-difluorovinyl)-4-fluorobenzamide (3h) ^1^H NMR**

**N-(2,2-difluorovinyl)-4-fluorobenzamide (3h) ^13^C NMR**

**N-(2,2-difluorovinyl)-4-fluorobenzamide (3h) ^19^F NMR**

**N-(2,2-difluorovinyl)-4-(trifluoromethoxy)benzamide (3i) ^1^H NMR**

**N-(2,2-difluorovinyl)-4-(trifluoromethoxy)benzamide (3i) ^13^C NMR**

**N-(2,2-difluorovinyl)-4-(trifluoromethoxy)benzamide (3i) ^19^F NMR**

**N-(2,2-difluorovinyl)-2-(naphthalen-1-yl)acetamide (3j) ^1^H NMR**

**N-(2,2-difluorovinyl)-2-(naphthalen-1-yl)acetamide (3j) ^13^C NMR**

**N-(2,2-difluorovinyl)-2-(naphthalen-1-yl)acetamide (3j) ^19^F NMR**

**5-chloro-N-(2,2-difluorovinyl)thiophene-2-carboxamide (3k) ^1^H NMR**

**5-chloro-N-(2,2-difluorovinyl)thiophene-2-carboxamide (3k) ^13^C NMR**

**5-chloro-N-(2,2-difluorovinyl)thiophene-2-carboxamide (3k) ^19^F NMR**

**5-fluoro-2-(naphthalen-1-yl)oxazole (4a) ^1^H NMR**

**5-fluoro-2-(naphthalen-1-yl)oxazole (4a) ^13^C NMR**

**5-fluoro-2-(naphthalen-1-yl)oxazole (4a) ^19^F NMR**

**5-fluoro-2-(naphthalen-2-yl)oxazole (4b) ^1^H NMR**

**5-fluoro-2-(naphthalen-2-yl)oxazole (4b) ^13^C NMR**

**5-fluoro-2-(naphthalen-2-yl)oxazole (4b) ^19^F NMR**

**5-fluoro-2-phenyloxazole (4c) ^1^H NMR**

**5-fluoro-2-phenyloxazole (4c) ^13^C NMR**

**5-fluoro-2-phenyloxazole (4c) ^19^F NMR**

**2-(4-(tert-butyl)phenyl)-5-fluorooxazole (4d) ^1^H NMR**

**2-(4-(tert-butyl)phenyl)-5-fluorooxazole (4d) ^13^C NMR**

**2-(4-(tert-butyl)phenyl)-5-fluorooxazole (4d) ^19^F NMR**

**5-fluoro-2-(4-methoxyphenyl)oxazole (4e) ^1^H NMR**

**5-fluoro-2-(4-methoxyphenyl)oxazole (4e) ^13^C NMR**

**5-fluoro-2-(4-methoxyphenyl)oxazole (4e) ^19^F NMR**

**5-fluoro-2-(p-tolyl)oxazole (4f) ^1^H NMR**

**5-fluoro-2-(p-tolyl)oxazole (4f) ^13^C NMR**

**5-fluoro-2-(p-tolyl)oxazole (4f) ^19^F NMR**

**5-fluoro-2-(o-tolyl)oxazole (4g) ^1^H NMR**

**5-fluoro-2-(o-tolyl)oxazole (4g) ^13^C NMR**

**5-fluoro-2-(o-tolyl)oxazole (4g) ^19^F NMR**

**5-fluoro-2-(m-tolyl)oxazole (4h) ^1^H NMR**

**5-fluoro-2-(m-tolyl)oxazole (4h) ^13^C NMR**

**5-fluoro-2-(m-tolyl)oxazole (4h) ^19^F NMR**

**5-fluoro-2-(4-(trifluoromethyl)phenyl)oxazole (4i) ^1^H NMR**

**5-fluoro-2-(4-(trifluoromethyl)phenyl)oxazole (4i) ^13^C NMR**

**5-fluoro-2-(4-(trifluoromethyl)phenyl)oxazole (4i) ^19^F NMR**

**methyl 4-(5-fluorooxazol-2-yl)benzoate (4j) ^1^H NMR**

**methyl 4-(5-fluorooxazol-2-yl)benzoate (4j) ^13^C NMR**

**methyl 4-(5-fluorooxazol-2-yl)benzoate (4j) ^19^F NMR**

**5-fluoro-2-(4-fluorophenyl)oxazole (4k) ^1^H NMR**

**5-fluoro-2-(4-fluorophenyl)oxazole (4k) ^13^C NMR**

**5-fluoro-2-(4-fluorophenyl)oxazole (4k) ^19^F NMR**

**5-fluoro-2-(4-(trifluoromethoxy)phenyl)oxazole (4l) ^1^H NMR**

**5-fluoro-2-(4-(trifluoromethoxy)phenyl)oxazole (4l) ^13^C NMR**

**5-fluoro-2-(4-(trifluoromethoxy)phenyl)oxazole (4l) ^19^F NMR**

**2-(4-chlorophenyl)-5-fluorooxazole (4m) ^1^H NMR**

**2-(4-chlorophenyl)-5-fluorooxazole (4m) ^13^C NMR**

**2-(4-chlorophenyl)-5-fluorooxazole (4m) ^19^F NMR**

**2-(2-chlorophenyl)-5-fluorooxazole (4n) ^1^H NMR**

**2-(2-chlorophenyl)-5-fluorooxazole (4n) ^13^C NMR**

**2-(2-chlorophenyl)-5-fluorooxazole (4n) ^19^F NMR**

**2-(3-chlorophenyl)-5-fluorooxazole (4o) ^1^H NMR**

**2-(3-chlorophenyl)-5-fluorooxazole (4o) ^13^C NMR**

**2-(3-chlorophenyl)-5-fluorooxazole (4o) ^19^F NMR**

**2-(3-bromophenyl)-5-fluorooxazole (4p) ^1^H NMR**

**2-(3-bromophenyl)-5-fluorooxazole (4p) ^13^C NMR**

**2-(3-bromophenyl)-5-fluorooxazole (4p) ^19^F NMR**

**5-fluoro-2-(3-iodophenyl)oxazole (4q) ^1^H NMR**

**5-fluoro-2-(3-iodophenyl)oxazole (4q) ^13^C NMR**

**5-fluoro-2-(3-iodophenyl)oxazole (4q) ^19^F NMR**

**5-fluoro-2-(3-nitrophenyl)oxazole (4r) ^1^H NMR**

**5-fluoro-2-(3-nitrophenyl)oxazole (4r) ^13^C NMR**

**5-fluoro-2-(3-nitrophenyl)oxazole (4r) ^19^F NMR**

**2-([1,1'-biphenyl]-2-yl)-5-fluorooxazole (4s) ^1^H NMR**

**2-([1,1'-biphenyl]-2-yl)-5-fluorooxazole (4s) ^13^C NMR**

**2-([1,1'-biphenyl]-2-yl)-5-fluorooxazole (4s) ^19^F NMR**

**2-(3,4-dimethylphenyl)-5-fluorooxazole (4t) ^1^H NMR**

**2-(3,4-dimethylphenyl)-5-fluorooxazole (4t) ^13^C NMR**

**2-(3,4-dimethylphenyl)-5-fluorooxazole (4t) ^19^F NMR**

**2-(3,5-dichlorophenyl)-5-fluorooxazole (4u) ^1^H NMR**

**2-(3,5-dichlorophenyl)-5-fluorooxazole (4u) ^13^C NMR**

**2-(3,5-dichlorophenyl)-5-fluorooxazole (4u) ^19^F NMR**

**1-(4-bromophenyl)-2,2-difluoroaziridine (5a) ^1^H NMR**

**1-(4-bromophenyl)-2,2-difluoroaziridine (5a) ^13^C NMR**

**1-(4-bromophenyl)-2,2-difluoroaziridine (5a) ^19^F NMR**

**2,2-difluoro-1-(naphthalen-2-yl)aziridine (5b) ^1^H NMR**

**2,2-difluoro-1-(naphthalen-2-yl)aziridine (5b) ^13^C NMR**

**2,2-difluoro-1-(naphthalen-2-yl)aziridine (5b) ^19^F NMR**

**2,2-difluoro-1-tritylaziridine (5c) ^1^H NMR**

**2,2-difluoro-1-tritylaziridine (5c) ^13^C NMR**

**2,2-difluoro-1-tritylaziridine (5c) ^19^F NMR**

**1-([1,1'-biphenyl]-4-yl)-2,2-difluoroaziridine (5d) ^1^H NMR**

**1-([1,1'-biphenyl]-4-yl)-2,2-difluoroaziridine (5d) ^13^C NMR**

**1-([1,1'-biphenyl]-4-yl)-2,2-difluoroaziridine (5d) ^19^F NMR**
